# PDCD10 Is a Key Player in TMZ-Resistance and Tumor Cell Regrowth: Insights into Its Underlying Mechanism in Glioblastoma Cells

**DOI:** 10.3390/cells13171442

**Published:** 2024-08-28

**Authors:** Yuan Zhu, Su Na Kim, Zhong-Rong Chen, Rainer Will, Rong-De Zhong, Philipp Dammann, Ulrich Sure

**Affiliations:** 1 Department of Neurosurgery and Spine Surgery, University Hospital Essen, University of Duisburg-Essen, 45147 Essen, Germany; suna.kim@outlook.de (S.N.K.); zhong-rong.chen@uk-essen.de (Z.-R.C.); zhongrongde@gmail.com (R.-D.Z.); philipp.dammann@uk-essen.de (P.D.); ulrich.sure@uk-essen.de (U.S.); 2 Center for Translational Neuro- and Behavioral Sciences (C-TNBS), University Hospital Essen, University of Duisburg-Essen, 45147 Essen, Germany; 3 Core Facility Cellular Tools, German Cancer Research Center (DKFZ), 69120 Heidelberg, Germany; r.will@dkfz-heidelberg.de

**Keywords:** glioblastoma (GBM), acquired TMZ-resistance, programmed cell death 10 (PDCD10), MGMT and MMR genes, stemness

## Abstract

Overcoming temozolomide (TMZ)-resistance is a major challenge in glioblastoma therapy. Therefore, identifying the key molecular player in chemo-resistance becomes urgent. We previously reported the downregulation of PDCD10 in primary glioblastoma patients and its tumor suppressor-like function in glioblastoma cells. Here, we demonstrate that the loss of PDCD10 causes a significant TMZ-resistance during treatment and promotes a rapid regrowth of tumor cells after treatment. PDCD10 knockdown upregulated MGMT, a key enzyme mediating chemo-resistance in glioblastoma, accompanied by increased expression of DNA mismatch repair genes, and enabled tumor cells to evade TMZ-induced cell-cycle arrest. These findings were confirmed in independent models of PDCD10 overexpressing cells. Furthermore, PDCD10 downregulation led to the dedifferentiation of glioblastoma cells, as evidenced by increased clonogenic growth, the upregulation of glioblastoma stem cell (GSC) markers, and enhanced neurosphere formation capacity. GSCs derived from PDCD10 knockdown cells displayed stronger TMZ-resistance and regrowth potency, compared to their parental counterparts, indicating that PDCD10-induced stemness may independently contribute to tumor malignancy. These data provide evidence for a dual role of PDCD10 in tumor suppression by controlling both chemo-resistance and dedifferentiation, and highlight PDCD10 as a potential prognostic marker and target for combination therapy with TMZ in glioblastoma.

## 1. Introduction

Glioblastoma (GBM) is the most common and aggressive malignant primary brain tumor in adults [1]. Despite established standard therapy including surgical treatment followed by radiation and adjuvant chemotherapy, tumor recurrence is currently unavoidable, and the overall survival period of patients is less than two years [2,3,4]. Acquired therapy resistance is a major determinant of recurrence.

Temozolomide (TMZ), an oral alkylating agent, is the first-line drug for glioblastoma chemotherapy, causing DNA damage and consequently tumor cell death [5,6]. However, the development of TMZ-resistance in glioblastoma patients limits treatment efficacy and results in rapid tumor regrowth [5,6,7]. TMZ-resistance can be acquired adaptively through the failure of DNA repair mechanisms and the enrichment of pre-existing therapy-resistant subclones. These subclones overtake the majority of malignant tissue, resulting in lethal recurrence [6,8,9]. This is exemplified by the association between decreased expression of DNA mismatch repair (MMR) genes and TMZ-resistance in recurrent GBM [10,11,12,13]. Another aspect contributing to TMZ-resistance is the presence of undifferentiated glioblastoma stem cells (GSCs) within the malignant tissue, which can increase the pool of resistant clones under TMZ pressure through genetic and epigenetic evolution [7,9,14]. To study the mechanisms behind acquired TMZ-resistance, we previously established a dedicated cell culture model [13], recapitulating in vivo TMZ-resistant features, such as sustained regrowth under/after TMZ treatment, downregulation of MMR genes, and increased stemness.

Programmed cell death 10 (PDCD10) is an adaptor protein that plays various roles in diverse biological processes, such as cell cycle, apoptosis, cell–cell junction, migration/invasion, angiogenesis, and vasculogenesis [15,16,17]. As its name implies, PDCD10 was first identified as a pro-apoptotic gene due to its upregulation upon apoptotic stimuli [18]. In cancers, PDCD10 shows multiface functions in a context-dependent manner [15,19,20,21,22,23,24]. In colon and breast cancer, PDCD10 deficiency confers chemotherapy-resistance [22,25]. We previously reported a downregulation of PDCD10 in 85% of a cohort of primary glioblastoma patients. An immunohistochemistry study revealed that PDCD10 was absent in the majority of tumor cells and in tumor endothelial cells, which was correlated inversely to tumor cell proliferation and hyperangiogenesis [26]. In addition to these findings from the immunohistochemistry study in GBM patient sections, we further demonstrated that a knockdown of PDCD10 in glioblastoma cells promoted malignant behaviors of tumor cells in vitro and stimulated tumor growth in vivo by triggering EphB4 kinase [21]. Moreover, a loss of PDCD10 in endothelial cells activated GBM cells via a paracrine mechanism, demonstrating its central role in the crosstalk between tumor cells and endothelial cells. We also defined PDCD10 as a key player in TMZ-resistance. PDCD10 knockdown induced apoptosis-resistance in vitro and chemotherapy-resistance in a pre-clinical model involving the inhibition of caspase-3 activation [20]. These findings established PDCD10 as a tumor suppressor-like factor in glioblastoma, and triggered our strong interest in gaining detailed insights into the underlying mechanism of PDCD10-dependent TMZ sensitivity using our established, acute, acquired TMZ-resistance model as a basis [13]. The acquired TMZ model allows us to study TMZ-resistant cells in response to new challenges, and is therefore highly relevant to TMZ therapy in the clinic.

## 2. Materials and Methods

### 2.1. Generation and Culture of PDCD10 Knockdown GBM Cells

PDCD10 was knocked-down by the lentiviral transduction of shRNA (shPDCD10) in two *human* GBM cell lines, U87 and T98g, as described previously [20]. Briefly, two different vector systems were used to knock-down PDCD10. For reversible knock-down in U87 (shU87) cells, a Doxycycline-inducible TRIPZ lentiviral shRNA vector for *human PDCD10* (shPDCD10; Thermo Scientific, Waltham, MA, USA, clone ID: V2THS_217165) was used. For constitutive knockdown in T98g cells (shT98g), a lentiviral shRNA vector for *human PDCD10* (OriGene, Rockville, MD, USA, cat# TL302576) was used. Red- and green-fluorescence proteins (RFP and GFP, respectively) were used as representative transduction markers. Empty vector transduced cells in U87 (evU87) (Thermo Scientific, Waltham, MA, USA, cat# RHS4750) and T98g (evT98g) (OriGene, Rockville, MD, USA, cat# TR30021) served as controls. Unless otherwise stated, transduced cells were maintained in Dulbecco’s modified Eagle’s medium (DMEM) growth medium, supplemented with 10% fetal bovine serum (FBS), 1% sodium pyruvate, and puromycin (1 µg/mL) (Sigma, Munich, Germany, cat# P8833-25 mg). For the inducible knockdown system in ev- and shU87, DMEM growth medium was additionally supplemented with Doxycycline (dox, 1 µg/mL) (Sigma, Munich, Germany, cat# D9891-10G). Transduction efficiency of ev/shGBM was monitored by flow cytometry or fluorescence microscopy, and PDCD10 expression was quantified by RT^2^-PCR and western blot.

### 2.2. Generation and Culture of Lentiviral Transduced PDCD10-Overexpressing GBM Cells

For the generation of stable overexpression cell lines of *human PDCD10* (oxGBM), U87 and T98g cells were transduced with a lentiviral vector (ox; pLX304_PDCD10; Core Facility Cellular tools, DKFZ, Heidelberg, Germany). Briefly, the open reading frame (ORF) of *human PDCD10* was shuttled into the lentiviral expression vector pLX304 (gift from David Root, Addgene, Cambridge, MA, USA, plasmid # 25890) containing a C-terminal V5 tag by use of gateway recombination technology (ThermoFisher, Braunschweig, Germany). Lentiviral particles were produced using a standard protocol. Briefly, HEK293FT (ThermoFisher, Braunschweig, Germany) cells were co-transfected with pLX304_PDCD10 and 2nd-generation viral packaging plasmids VSV.G (Addgene, Cambridge, MA, USA; plasmid #14888) and psPAX2 (Addgene; plasmid #12260). The medium was changed to the respective cell culture medium without antibiotics 24 h after transfection. After an additional 24 h, the viral supernatant was collected and filtered through a 0.45 µm filter. U87- and T98g cells were transduced for 24 h in 6-well plates with different dilutions (1 mL, 100 µL, 10 µL, 1 µL, 0.1 µL, 0) of viral supernatant in the presence of 10 µg/mL polybrene. After viral clearance, transduced cells were selected in DMEM growth medium supplemented with either 5 or 3 µg/mL blasticidin (Gibco, Darmstadt, Germany, cat# A1113902) for U87 and T98g, respectively. In the wells with fewer than 20 colonies, single colonies were picked and expanded. As a control, pLX304 empty vector-transduced cells in U87 and T98g were employed (evGBM). Stable overexpression after passaging was verified by RT^2^-PCR for PDCD10 and by western blot for both PDCD10 and V5.

### 2.3. Generation of TMZ-Resistant Variants in ev/shPDCD10-GBM Cells

Acquired TMZ-resistant cells were generated in ev/shPDCD10-GBM cells (ev/shGBM), as described in our recently published paper with modifications [13]. Briefly, based on their sensitivity to TMZ [20], GBM cell lines were either treated with 150 µM for ev/shU87 cells, or 300 µM for ev/shT98g of TMZ (Sigma, Munich, Germany, cat# T2577). Damaged cells and TMZ were washed out 72 h after the treatment, and the remaining viable cells were cultured in TMZ-free medium for 72 h to allow regrowth, and then subjected to the second cycle of treatment for 72 h and washing. The viable cells regrew in normal culture medium and were defined as regrown (RG) TMZ-resistant cells (ev/shRG). Transduction efficiency of ev- and shRG was controlled by flow cytometry or fluorescence microscopy, and knockdown of PDCD10 in shRG cells was evaluated by RT^2^-PCR.

### 2.4. Real-Time RT-PCR (RT^2^-PCR)

Total RNA was isolated using the innuPREP DNA/RNA Mini Kit (Analytic Jena AG, Jena, Germany, cat# 845-KS-2040050). cDNA was synthesized using iScript cDNA Synthesis Kit (Bio-rad, Munich, Germany, cat# 1708891), according to the manufacture’s manual. RT^2^-PCR was performed using 2× qPCRBIO SyGreen Mix with Fluorescein (PCRBIOSYSTEMS, London, UK, cat# PB20.13-05). According to our established protocol [13], the PCR program was set as follows: initial denaturation at 95 °C for 2 min, 40 cycles of amplification at 95 °C for 5 s, and at annealing temperature for 25 s. A melting curve analysis was included with the following setting: 95 °C for 1 min, 55–95 °C with a heating increase rate of 0.5 °C every 10 s. Primers and corresponding annealing temperatures used in the present study are listed in Table 1. The relative expression of a gene of interest was calculated by the 2^−ΔΔCt^ method with a reference gene, *GAPDH* or *RPS13*, for U87 or T98g, respectively.

### 2.5. Western Blot

Total protein extraction and western blot were performed, as described previously [13]. The following antibodies were used: *rabbit* anti-PDCD10 (abcam, Cambridge, UK, cat# ab180706; 1:800), *mouse* anti-V5 (Invitrogen, Darmstadt, Germany, cat# R960-25; 1:2500), *rabbit* anti-MGMT (Cell Signaling Technology, Danvers, MA, USA, cat# 2739; 1:1000), *rabbit* anti-GAPDH (Cell Signaling Technology, Danvers, MA, USA; cat# 2118; 1:1000), *goat* anti-*rabbit* IgG, HRP-linked (cell signaling; cat# 7074; 1:2000), and *horse* anti-*mouse* IgG, HRP-linked (Cell Signaling Technology, Danvers, MA, USA; cat# 7076s; 1:1000). Signal was detected by chemiluminescence using ECL substrates (Clarity, Bio-rad, Hercules, CA, USA, cat# 1705060) and ImageQuant LAS 500 (GE Healthcare, Freiburg, Germany).

### 2.6. Evaluation of Cell Viability in TMZ Treatment Phase and in Post-Treatment Phase by MTT Assay

To study the roles of PDCD10 in TMZ-resistance, cell viability was detected by MTT assay in ev/shGBM cells, ev/shRG cells, and ev/oxGBM cells after 72 h of TMZ treatment (treatment phase) and in the post-treatment phase. Two models of post-treatment were applied: (i) living cells continuously regrew (without reseeding) after washing-out treated TMZ; (ii) living cells were reseeded at identical density after washing-out TMZ, and were cultured for 3 d in drug-free medium. Transduced U87 and T98g cells were treated with 150 µM and 300 µM of TMZ, respectively. The control cells (C) received the treatment of vehicle dimethyl sulfoxide (DMSO) (Sigma, D8418) at the concentrations of 0.1% and 0.2% DMSO for U87 and T98g cells, respectively.

MTT assay (Invitrogen, Darmstadt, Germany, cat# M6494) was carried out, as described previously [20]. Briefly, 10 µL MTT reagent was added to each well and incubated for 3 h at 37 °C and 5% CO_2_. To lyse cells and dissolve intracellular formazan crystals, 50 µL of pure DMSO was added to each well after removing the medium containing MTT. The absorbance of formazan was measured using a microplate reader (Infinite 200 Pro, Tecan, Männedorf, Switzerland) at 540 nm, and at 630 nm as a reference.

### 2.7. EdU Proliferation Assay and Cell Cycle Assay by Flow Cytometry

To detect DNA-replicating cells, 5-ethynyl-2’-deoxyuridine (EdU) incorporation was performed, as described previously [13]. Briefly, cells were incubated in fresh DMEM growth medium supplemented with 15 µM EdU (Lumiprobe, Hannover, Germany, cat# 10540) for 1 h at 37 °C and 5% CO_2_. Cells were harvested, fixed, and permeabilized in 0.1% Triton-X-100 in PBS for 30 min at room temperature. For click reaction to label and detect incorporated EdU with fluorescence Cy5, cells were incubated with a reaction cocktail containing 3 µM sulfo-Cy5-azide (Lumiprobe, Hannover, Germany, cat# A3330), 2 mM CuSO_4_ (Sigma, cat# 209198), and 20 mg/mL ascorbic acid (Sigma, cat# A4544) in 100 mM Tris-PBS (pH 7.6) for 30 min at room temperature. Cell nuclei were stained with DAPI (1.5 mg/mL; Sigma, Munich, Germany, cat# D8418) in 0.1% Triton-X-100 in PBS for 30 min at room temperature.

For flow cytometry (FACS), EdU-incorporated cells and DNA-stained cells were detected using a CytoFLEX instrument (Beckman Coulter, Indianapolis, IN, USA) with the setting as follows: 20,000 events for the record and 10,000 events for display. Cell cycle distribution was defined using FlowJo (v10.8, BD Life Sciences, Ashland, OR, USA) with the Dean–Jett–Fox algorithm.

### 2.8. Colony Formation Assay

ev/shT98g-RG and ev/oxT98g cells (1 × 10^5^/well) were seeded in a 12-well plate and treated with TMZ or with DMSO. After 72 h of incubation, cells were used to set up a colony formation assay (CFA), as described previously [13]. Briefly, cells were harvested and reseeded in a 12-well plate at densities of 500 cells for ev/shT98g-RG and 1000 cells for ev/oxT98g, followed by 13 d of incubation. The growth medium was refreshed after 5–6 d of incubation. Thereafter, cells were fixed in 4% paraformaldehyde and stained with 0.5% crystal violet. Stained colonies were imaged using a digital scanner. The number of colonies per well were analyzed using the ImageJ software (version 1.54j) [27].

### 2.9. Neurosphere Assay and Characterization of Glioblastoma Stem Cells (GSC)

To evaluate the self-renewal capacity of cells, a neurosphere assay (NSA) was performed. U87-RG (200 cells/well) were seeded in a 96-well plate and cultured in serum-free stem cell culture medium: DMEM;F12 (Gibco, cat# 11320033) was supplemented with 1× B27 (Gibco, cat# 17504044), 2× N2 (Gibco, cat# 17502048), 20 ng/mL recombinant *human* EGF (Peprotech, Hamburg, Germany; cat# AF-100-15), and 20 ng/mL recombinant *human* FGF-basic (Peprotech; cat# 100-18C). For the culture of ev- and shU87-RG cells, the medium was additionally supplemented with 1 µg/mL doxycycline and 1 µg/mL puromycin. The serum-free medium was half-refreshed every 3–4 d. Spheres were cultured for around 2 wk until spheres became larger than 50 µm in diameter and imaged using the LAS X software (3.7.6.25997, Leica, Wetzlar, Germany) with a 5× objective. The size of individual spheres was measured using the LAS X software (3.7.6.25997, Leica, Wetzlar, Germany). Spheres larger than 50 µm in diameter were counted. Sphere formation efficiency (SFE, %) was calculated with the following formula: (number of spheres/number of seeded cells per well) × 100.

To enrich stem-like cells from U87-RG (ev/shU87-RG-GSC), ev- and shRG cells (5 × 10^5^) were seeded in a 60 mm PD and cultured in serum-free medium, prepared as described above. Serum-free media were refreshed every 3–5 d until spheres reached over 100 µm in diameter. After up to 2 wk of incubation, sphere-forming cells in suspension were dissociated into single cells using StemPro Accutase (Gibco, cat# A11105-1) and were either passaged for further expansion in serum-free medium or subjected to individual experiments.

To assess the susceptibility of shRG-GSC to TMZ treatment, cells (3000/well) were seeded in a 96-well plate coated with poly-l-lysine (Sigma, cat# P1274) to enhance cell adherence. After overnight incubation in serum-free medium, cells were treated with TMZ (150 µM) for 72 h. The viability of RG-GSC variants was evaluated using Orangu (Cell guidance systems Ltd., Cambridge, UK, cat# OR01-500), an alternative to MTT, which utilizes WST-8-producing water-soluble tetrazolium salt and is more suitable for sensitive cells such as GSCs.

### 2.10. Statistics

Statistical analysis was performed using GraphPad Prism 9. Data are presented as mean and standard deviation (mean ± s.d.) unless otherwise stated. Differences between two and multiple groups were analyzed by unpaired *t*-test and by two-way ANOVA with the Sidak method for multiple comparisons, respectively. A *p* value < 0.05 was considered statistically significant.

## 3. Results

### 3.1. PDCD10 Knockdown in GBM Cells Leads to TMZ-Resistance and Tumor Cell Regrowth

**Figure 1:**
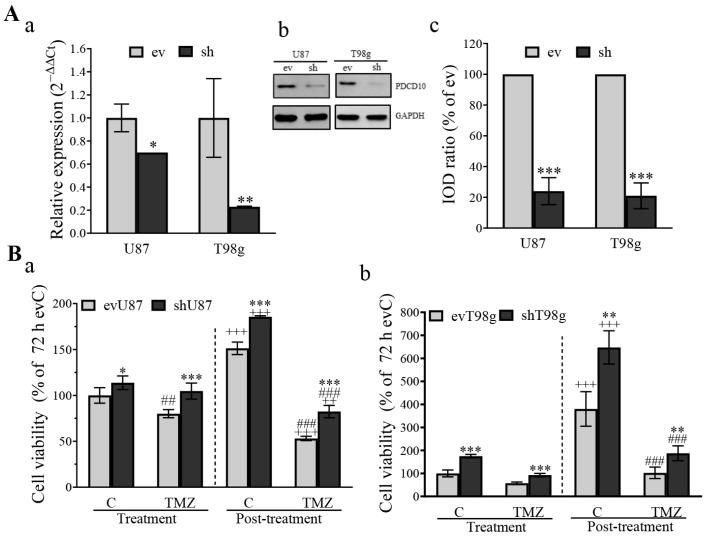
Knockdown of PDCD10 confers TMZ-resistance on GBM cells. (**A**) Confirmation of PDCD10 knockdown in lentiviral transduced U87 and T98g cells by RT^2^-PCR (**a**), western blot (**b**), and semi-quantitation of the blots (**c**). ev and sh: empty vector- and PDCD10 shRNA-transduced cells, respectively. IOD: integrated optical density. *, *p* < 0.05; **, *p* < 0.01; ***, *p* < 0.001, compared with ev. (**B**) Knockdown of PDCD10 in GBM cells leads to a resistance to TMZ-induced cell death. U87 and T98g cells received the treatment with 150 µM (**a**) and 300 µM (**b**) of TMZ, respectively, for 72 h. Thereafter, TMZ was washed-out. Remaining viable cells were cultured in the TMZ-free medium for 3 d, which is defined as the post-treatment phase. Control cells (C) were treated with vehicle DMSO (0.1% and 0.2% for U87 and T98g, respectively). MTT assay was performed to determine the viability of cells at 72 h after TMZ treatment (treatment phase) and 3 d after washing-out of TMZ (post-treatment phase). *, *p* < 0.05; **, *p* < 0.01; ***, *p* < 0.001, compared with ev; ##, *p* < 0.01; ###, *p* < 0.0001, compared with evC in the same phase; +++, *p* < 0.001, compared with corresponding group in treatment phase.

Before using transduced GBM cells for individual experiments, the knockdown in parental shU87 and shT98g was monitored at both mRNA and protein levels by RT^2^-PCR (Figure 1Aa) and by western blot (Figure 1Ab,c).

For proof-of-concept, we first evaluated cell viability in parental cells in response to TMZ treatment. shPDCD10 significantly enhanced cell viability under control conditions (C) and 72 h after TMZ treatment (treatment phase) in both U87 (Figure 1Ba) and T98g (Figure 1Bb). Moreover, a rapid regrowth of shU87 and shT98g cells was detected 3 d after regrowth in TMZ-free medium (post-treatment phase), compared with the corresponding ev cells. These data demonstrate that the loss of PDCD10 in GBM cells promotes cell proliferation in both the absence and presence of TMZ, and leads to rapid regrowth during the post-treatment phase.

### 3.2. PDCD10 Knockdown in RG Cells Increases Cell Viability during TMZ Treatment and Restores Growth Capacity in the Post-Treatment Phase

**Figure 2:**
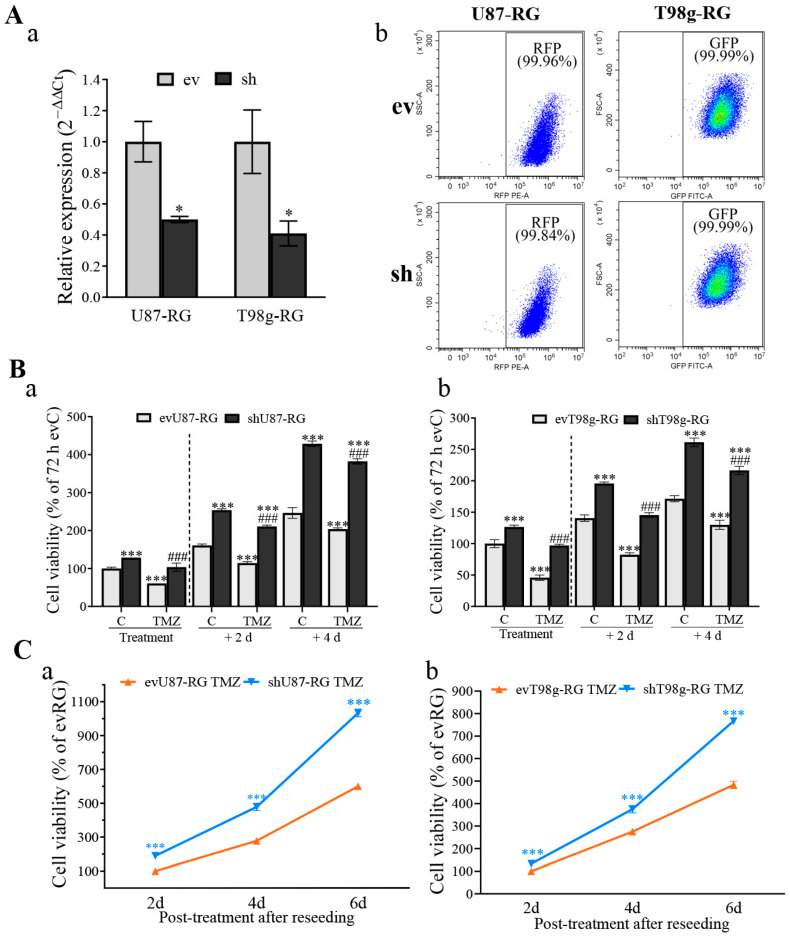
Knockdown of PDCD10 enhances cell viability after rechallenge with TMZ in regrown cells (RG) generated from the established acquired TMZ-resistant model. (**A**) Confirmation of PDCD10 knockdown in shU87-RG and shT98g-RG cells by RT^2^-PCR (**a**) and by FACS of respective transduced cells that expressed red-fluorescence protein (RFP) and green-fluorescence protein (GFP) (**b**). *, *p* < 0.05, compared with ev. (**B**) MTT assay in RG cells in treatment phase and post-treatment phase. MTT assay was performed with ev/shU87 and ev/shT98g cells that received the treatment with 150 µM (**a**) and 300 µM (**b**) of TMZ, respectively, for 72 h (treatment phase) and 2 d and 4 d after washing-out TMZ (post-treatment phase, without reseeding). Control cells (C) received vehicle DMSO (0.1% and 0.2% for U87 and T98g, respectively). *, *p* < 0.05; ***, *p* < 0.001, compared with evRG C (72 h); ###, *p* < 0.001, compared with evRG-TMZ (72 h). (**C**) MTT assay in RG cells in a second post-treatment model with reseeding. ev/shU87-RG and ev/shT98g-RG cells received the treatment with 150 µM (**a**) and 300 µM (**b**) of TMZ, respectively, for 72 h. Thereafter, TMZ–containing media and dead cells were washed-out, and the viable cells were harvested and reseeded at the same density, followed by 2, 4, and 6 d of culture in drug-free medium. A significantly more rapid regrowth was observed in both TMZ-treated shU87-RG and shT98g-RG cells, compared with the corresponding evRG cells after reseeding and culturing in drug-free media. *, *p* < 0.05; ***, *p* < 0.001, compared with corresponding evRG.

Next, we investigated the role of PDCD10 on TMZ-resistance in resistant (regrown, RG) cells generated from two cycles of TMZ treatment. The transduction efficiency in ev/shRG cells was confirmed by RT^2^-PCR analysis (Figure 2Aa) and by FACS analysis for RFP and GFP in transduced U87-RG and T98g-RG cells, respectively (Figure 2Ab).

To evaluate the acquired TMZ-resistance and subsequent regrowth capacity of RG cells, an MTT assay was conducted after 72 h of TMZ treatment (treatment phase) and on day 2 and day 4 after washing-out TMZ (post-treatment phase). Similar to the observation in shGBM cells (Figure 1B), shU87-RG cells exhibited a significantly higher viability under control conditions (C) (*p* < 0.001) and in the TMZ treatment phase (*p* < 0.001), compared with the corresponding ev groups (Figure 2Ba). Moreover, shU87-RG cells showed a significantly rapid regrowth in the post-treatment phase compared with respective evU87-RG cells. Similar results were obtained in shT98g-RG cells (Figure 2Bb). Of note, the regrowth potential was much more pronounced in shRG cells (Figure 2B), compared with their parental cells (shGBM) (Figure 1B).

Along with these interesting findings from the post-treatment phase, we validated the restoration of cell growth using a second post-treatment model as follows: cells were harvested from evRG and shRG cultures after washing-out TMZ and were reseeded at an identical density. This was followed by culturing for 2, 4, and 6 d in a drug-free medium. As revealed by the MTT assay, both shU87-RG (Figure 2Ca) and shT98g-RG (Figure 2Cb) cells exhibited a significantly more rapid cell proliferation, compared with that in the corresponding evU87-RG and evT98g-RG cells at all time points during the post-treatment period (*p* < 0.001). These data highlight the potent restoration of regrowth capacity of shRG cells in the TMZ post-treatment phase. Thus, RG cells may serve as an appropriate in vitro model to study PDCD10 function in acquired TMZ-resistance.

### 3.3. Overexpression of PDCD10 Sensitizes GBM Cells to TMZ Treatment

**Figure 3:**
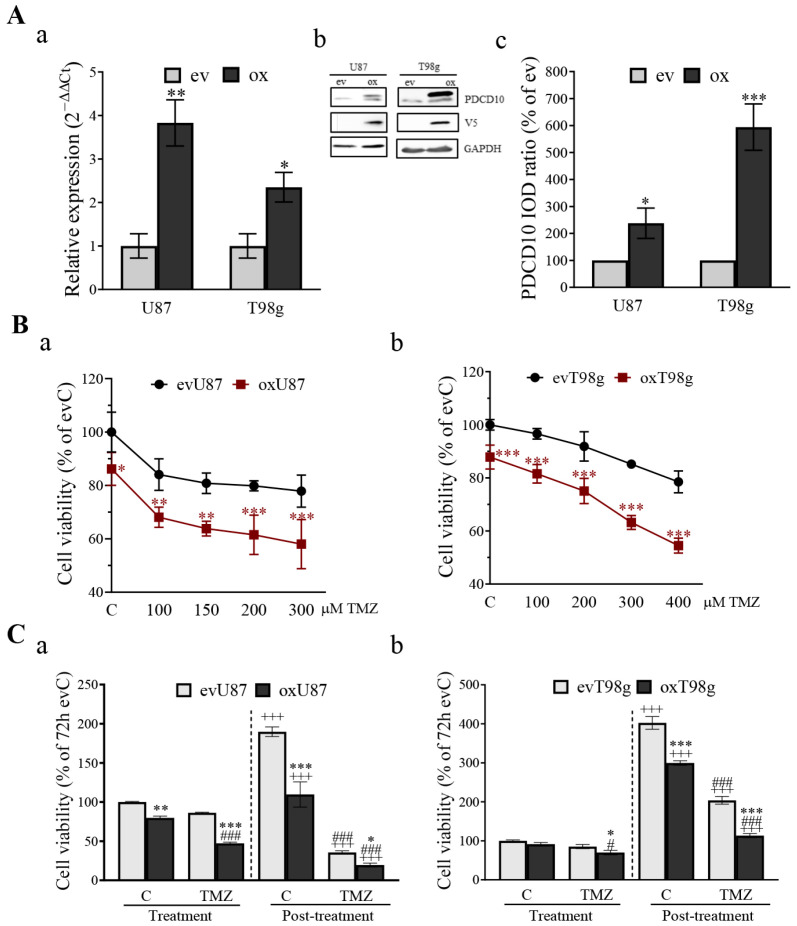
Overexpression of PDCD10 sensitizes GBM cells to TMZ treatment. (**A**) Confirmation of overexpression of PDCD10 in lentiviral transduced U87 and T98g cells by RT^2^-PCR (**a**) and western blot (**b**) and semi-quantitation of the blots (**c**). Western blotting with anti-V5 antibody distinguishes between the expression of transgenic C-terminal V5-tagged PDCD10 protein and endogenous protein. ev and ox: empty vector-transduced and PDCD10-overexpressing cells, respectively. IOD: integrated optical density. *, *p* < 0.05; **, *p* < 0.01; ***, *p* < 0.001, compared with ev. (**B**) Overexpression of PDCD10 significantly reduces cell viability in a concentration-dependent manner after 72 h of TMZ treatment in both oxU87 (**a**) and oxT98g (**b**) cells. Control cells (C) were treated with vehicle DMSO (0.1% and 0.2% for U87 and T98g, respectively). *, *p* < 0.05; **, *p* < 0.01; ***, *p* < 0.001, compared with corresponding ev groups. (**C**) Overexpression of PDCD10 sensitizes GBM cells to TMZ treatment 72 h after TMZ treatment (treatment phase) and 3 d after washing-out TMZ (post-treatment phase). ev/oxU87 and ev/oxT98g cells received the treatment with 150 µM (**a**) and 300 µM (**b**) of TMZ for 72 h, respectively. Thereafter, TMZ-containing medium and dead cells were washed-out and the viable cells were further cultured in drug-free medium for 3 d followed by MTT assay. Control cells (C) were treated with vehicle DMSO (0.1% and 0.2% for U87 and T98g, respectively). *, *p* < 0.05; **, *p* < 0.01; ***, *p* < 0.001, compared with corresponding ev groups; +++, *p* < 0.001, compared with corresponding evC in the treatment phase; #, *p* < 0.05, ###, *p* < 0.001, compared with corresponding evC in the same phase.

Next, we further validated the impact of PDCD10 on TMZ-induced cytotoxicity using PDCD10-overerexpressing GBM (oxGBM) cells. A 3.83-fold (*p* < 0.01) and 2.35-fold (*p* < 0.05) upregulation of PDCD10 mRNA expression was confirmed in oxU87 and oxT98g cells, respectively (Figure 3Aa). Western blot demonstrated the upregulated protein level of PDCD10 in both types of oxGBM cells (Figure 3Ab,c). MTT assay showed that cell susceptibility significantly increased in a dose-dependent manner after 72 h of TMZ treatment in both oxU87 and oxT98g cells, compared to their corresponding evGBM groups (Figure 3Ba,b). Due to the intrinsic TMZ-resistance associated with MGMT expression in T98g cells [28], a lower cell susceptibility was observed in ev/oxT98g cells than in ev/oxU87 cells at an identical concentration of TMZ.

Similar to the models used in ev/shGBM cells, we checked cell viability in both TMZ treatment and post-treatment phases in ev/oxGBM cells (Figure 3C). Overexpression of PDCD10 in U87 (oxU87) resulted in a 20.18% (*p* < 0.001) decrease in cell growth in the treatment phase, and a 42.22% (*p* < 0.001) reduction in cell regrowth in the post-treatment phase, compared to the corresponding evU87 cells under control conditions (C). Furthermore, a significant attenuated cell viability (45.20%, *p* < 0.001) was observed in oxU87 cells after 72 h of TMZ treatment (treatment phase), and suppressed cell regrowth (33.75%, *p* < 0.001) was detected at 3 d of culture in drug-free medium (post-treatment phase), compared to the respective ev groups. Notably, the viability of oxU87 cells decreased more significantly in the post-treatment phase than in the treatment phase (*p* < 0.001), suggesting that overexpressing PDCD10 largely prevented the restoration of GBM cell regrowth capacity in the post-treatment phase. Similar results were obtained from oxT98 cells (Figure 3Cb).

### 3.4. Alteration of DNA Replication and Cell Cycle in a PDCD10-Dependent Manner in Response to TMZ Treatment

**Figure 4:**
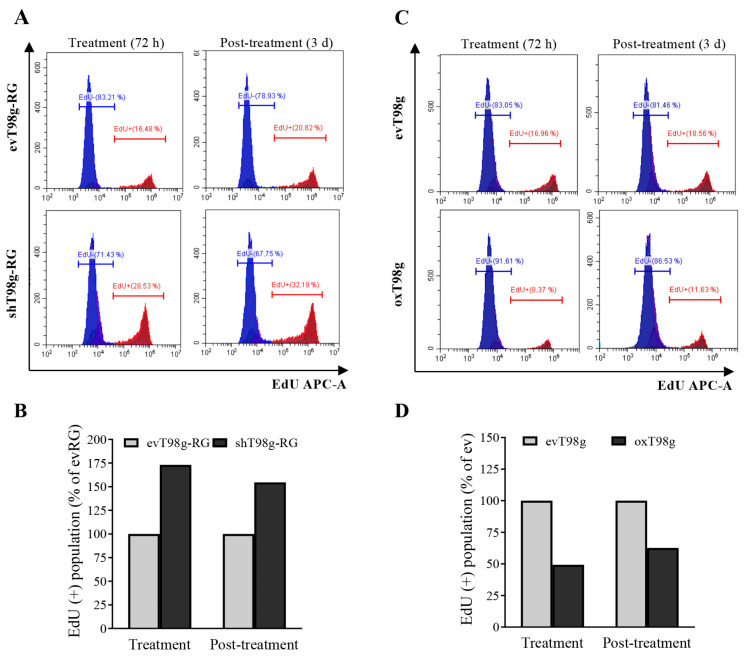
DNA replication in response to TMZ treatment is dependent on PDCD10 expression. DNA replication was detected by EdU incorporation followed by FACS at 72 h of TMZ treatment (treatment phase) and at 3 d after TMZ-washing-out and -culturing in drug-washout media (post-treatment phase). ev/shT98g-RG and ev/oxT98g cells received 500 and 300 µM TMZ, respectively. (**A**,**C**) Histograms of EdU-positive (EdU+) and -negative (EdU−) cell populations in ev/shT98g-RG and ev/oxT98g cells, respectively. (**B**,**D**) Bar graphs of EdU+/− populations based on the corresponding histograms in (**A**,**C**). Knockdown of PDCD10 leads to an increase in DNA replication in both the treatment and post-treatment phases of T98g-RG cells, whereas overexpression of PDCD10 suppresses DNA replication in response to TMZ treatment. The data are representative of at least three independent experiments.

PDCD10 knockdown increased cell viability and regrowth capacity during TMZ treatment (Figure 1Ac). Moreover, we observed stronger and faster regrowth of PDCD10 knockdown cells in the TMZ post-treatment phase (Figure 2). Overexpression of PDCD10 induced inverse effects. We therefore proceeded to investigate whether DNA replication was affected in these models by utilizing a 5-ethynyl-2′-deoxyuridine (EdU) incorporation assay. EdU-positive (EdU+) cells are representative of DNA-replicating cells. Figure 4A shows an EdU incorporation histogram in ev- and shT98g-RG cells after 72 h of TMZ treatment (treatment) and after 3 d of culture in TMZ-free medium (post-treatment). Further analysis revealed a 73.1% and 56.90% increase in DNA-replicating cells in PDCD10 knockdown cells during and after treatment, compared to corresponding controls (Figure 4B). In contrast, we observed a 50.7% and 37.3% reduction of DNA-replicating cells in PDCD10 overexpressing cells, compared to controls, in the TMZ treatment phase and post-treatment phase, respectively (Figure 4C,D).

**Figure 5:**
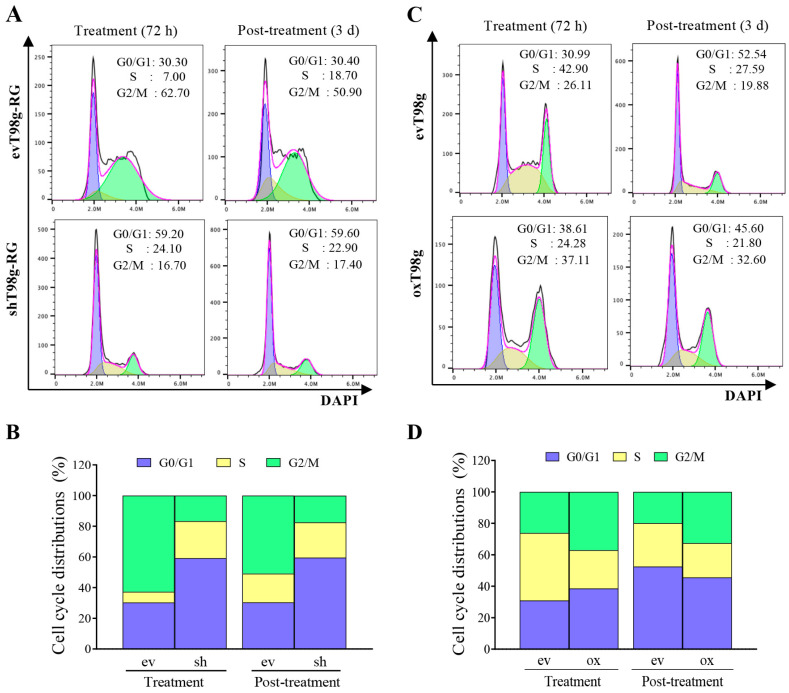
Alteration in cell cycle checkpoints in response to TMZ treatment is dependent on PDCD10 expression. Cell cycle assay was performed by FACS after 72 h of TMZ treatment (treatment phase) and at 3 d after TMZ-washing-out and -culturing in drug-washout media (post-treatment phase). ev/shT98g-RG and ev/oxT98g cells received 500 and 300 µM TMZ, respectively. (**A**,**C**) are representative of cell cycle histograms in ev/shT98g-RG and ev/oxT98g cells, respectively. DNA content-based cell cycle distributions were defined using FlowJo with the Dean–Jett–Fox algorithm and presented in histograms. Each cell cycle phase is shown in different colors: G0/G1- (blue), S- (yellow), and G2/M-phase (green). (**B**,**D**) Stacked bar graphs of the distribution of the cell population in the cell cycle based on the corresponding histograms in (**A**,**C**). Knockdown of PDCD10 leads to the escape of cells from G2/M arrest and increases the population in the S phase (DNA replication phase) in both the treatment and post-treatment phases, whereas overexpression does the opposite. The data are representative of at least three independent experiments.

To investigate the role of PDCD10 in cell cycle progression, cell cycle status in the TMZ treatment and post-treatment phases was assessed by FACS. Knockdown of PDCD10 led to an increase in the S phase, but a decrease in the G2/M phase in response to TMZ treatment (Figure 5A,B), whereas overexpression of PDCD10 produced the opposite effects (Figure 5C,D). These findings strongly corroborate the DNA replication data presented in Figure 4, and suggest that shRG cells evade DNA replication stalling and cell cycle arrest, allowing sustained growth despite TMZ treatment. This effect could be reversed by overexpression of PDCD10. Similar results were observed in shU87-RG cells (Figure S1). As a control, a cell cycle assay was also performed with vehicle-treated ev/shT98g-RG and ev/oxT98g. The histograms are presented in Figures S2 and S3. Cell cycle raw data, including sub-G1 phase, are shown in Figure S4.

### 3.5. PDCD10 Knockdown Upregulates MGMT and Downregulates the Expression of DNA Mismatch Repair (MMR) Genes

**Figure 6:**
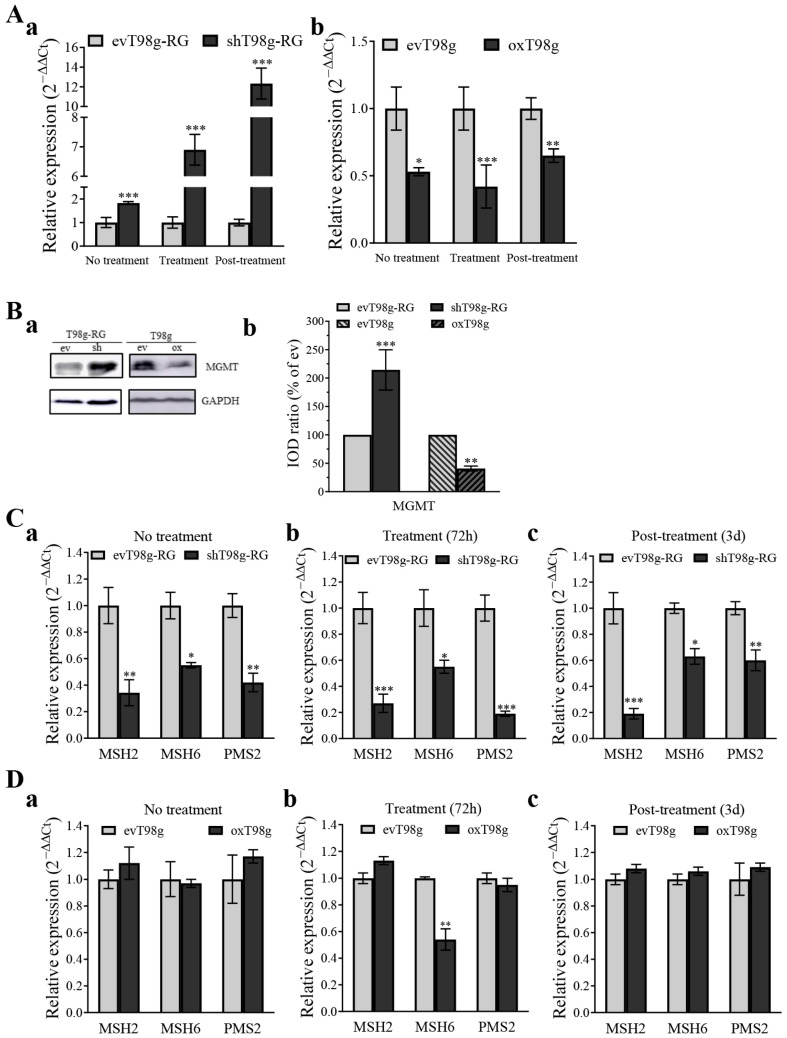
Knockdown of PDCD10 in T98g-RG cells leads to deregulation of DNA damage response (DDR) genes. ev/shT98g-RG and ev/oxT98g cells received 300 µM of TMZ or vehicle (0.2% DMSO) treatment (no TMZ treatment). Cells were harvested for PCR detection of DDR genes after 72 h of TMZ treatment (treatment phase) and at 3 d after TMZ-washing-out and culturing in drug-free media (post-treatment phase). (**A**) Expression of MGMT in T98g-RG (**a**) cells and ev/oxT98g (**b**) cells in the no treatment, treatment (300 µM, 72 h), and post-treatment (3 d after washing out) phases. (**B**) Western blot (**a**) and semi-quantitation of the blots (**b**) of the MGMT protein expression in ev/shT98g-RG and ev/oxT98g cells. (**C**) Expression of DDR genes (*MSH2*, *MSH6,* and *PMS2*) in T98g-RG cells in the no treatment (**a**), treatment (**b**), and post-treatment phases (**c**), respectively. (**D**) Expression of DDR genes in ev/oxT98g cells in the no treatment (**a**), treatment (**b**), and post-treatment phases (**c**), respectively. *, *p* < 0.05; **, *p* < 0.01 and ***, *p* < 0.001, compared with corresponding ev.

To investigate the mechanism of DNA repair and genomic instability in PDCD10 knockdown-induced TMZ-resistance, we detected the expression of MGMT and DNA mismatch repair (MMR) genes that play a pivotal role in the DNA damage response (DDR) and trigger cell cycle arrest upon TMZ treatment [5]. MGMT expression was notably upregulated in shT98g-RG cells, compared to in corresponding evT98g-RG cells across various conditions, including no treatment, TMZ treatment, and post-treatment phases (Figure 6Aa). In contrast, MGMT expression in oxT98g cells was downregulated across all three conditions mentioned above (Figure 6Ab). This finding was subsequently validated by western blot (Figure 6B). RT^2^-PCR revealed that the basal levels of MMR genes *MSH2*, *MSH6*, and *PMS2* were downregulated by 0.34-, 0.55-, and 0.62-fold, respectively, in shT98g-RG cells, compared to in evT98g-RG cells (Figure 6Ca). Furthermore, shT98g-RG cells exhibited a consistent downregulation of these MMR genes in the TMZ treatment (Figure 6Cb) and post-treatment (Figure 6Cc) phases. In contrast, oxT98g cells displayed little to no change in the expression of these MMR genes, compared to evT98g cells.

### 3.6. PDCD10 Knockdown Enhances Colony Formation Capacity of RG Cells

**Figure 7:**
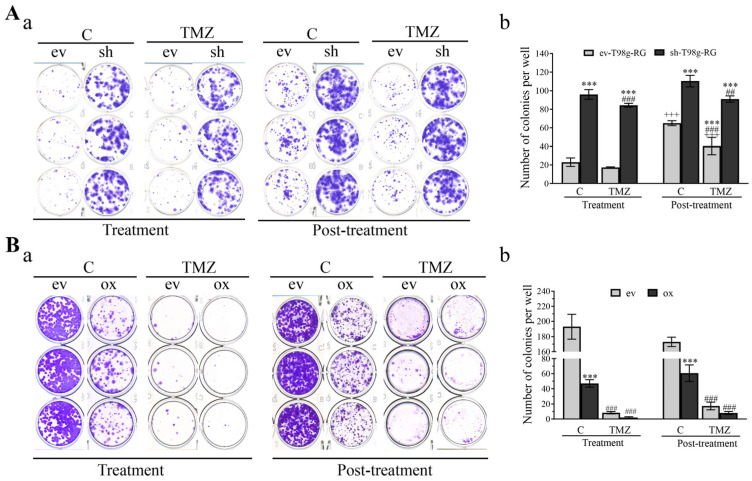
PDCD10 expression determines the colony formation capacity of GBM cells. ev/shT98g-RG (**A**) and ev/oxT98g (**B**) cells received 300 µM of TMZ or vehicle (0.2% DMSO) treatment. Cells were harvested for colony formation assay in a 12-well plate in triplicate after 72 h of TMZ treatment (treatment phase) and at 3 d after TMZ-washing-out and culturing in drug-free media (post-treatment phase). The number of colonies was quantified after staining with 0.5% crystal violet using the ImageJ software (version 1.54j). Representative images of colony formation in ev/shT98g-RG and ev/oxT98g are shown in (**Aa**) and (**Ba**), respectively. Quantitative analysis of the colony numbers is presented in (**Ab**) and (**Bb**) for ev/shT98g-RG and ev/oxT98g cells, respectively. ***, *p* < 0.001, compared with ev; ##, *p* < 0.01 and ###, *p* < 0.001, compared with corresponding C; +++, *p* < 0.001, compared with corresponding group in the treatment phase.

A colony formation assay was used to quantitatively examine the capability of single cells to grow into a large colony through clonal expansion in vitro. Clonogenic activity serves as a sensitive indicator of undifferentiated cancer stem cells, which represents a mechanism of resistance to TMZ [29]. Given that PDCD10 knockdown resulted in TMZ-resistance and promoted cell regrowth, we performed a colony assay using RG cells that survived TMZ treatment. Figure 7Aa,Ba show the representative images of colony formation in PDCD10-knockdown cells and PDCD10-overexpressing cells, respectively. Quantitative analysis (Figure 7Ab) revealed a 4.17- (*p* < 0.001) and 4.87-fold (*p* < 0.001) increase in colony numbers in shT98g-RG cells, compared to evT98g-RG cells under basal conditions (C) and 72 h after TMZ treatment, respectively. Interestingly, in the post-treatment phase, the colony formation increased in all groups to different extents, compared with the corresponding groups in the treatment phase, suggesting that the cells that survived the TMZ treatment quickly restored their tumorigenic potential in the post-treatment phase. The colony-formation ability of shT98g-RG cells increased 1.69-fold (*p* < 0.001) and 2.26-fold (*p* < 0.001), compared with corresponding evT98g-RG cells in the treatment and post-treatment phases, respectively. In contrast, overexpression of PDCD10 significantly diminished the colony formation not only under control conditions (C) but also in both the treatment and post-treatment phases (Figure 7Bb). These data raised a pivotal role for PDCD10 in tumor initiation/propagation, which could potentially be associated with its effects on TMZ-resistance.

### 3.7. PDCD10 Knockdown Enhances Stemness Properties of RG Cells in Association with TMZ-Resistance

**Figure 8:**
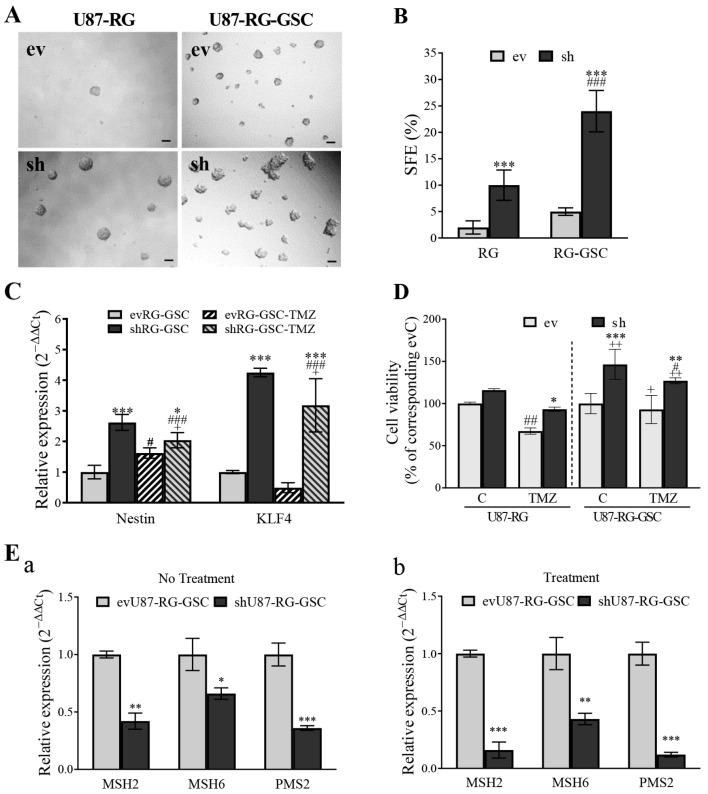
Knockdown of PDCD10 enhances the self-renewal capacity of U87-RG cells and GSCs generated from the parental cell line U87-RG (U87-RG-GSCs), and increases the expression of stem cell markers in RG-GSCs. (**A**) Representative images of neurospheres derived from U87-RG cells and U87-RG-GSCs. Scale bar: 100 µm. (**B**) Quantitative analysis of neurospheres formation efficiency (SFE). ***, *p* < 0.001, compared with corresponding ev; ###, *p* < 0.001, compared with corresponding parental cells. (**C**) Knockdown of PDCD10 increases the mRNA expression of stemness genes in RG-GSCs. The expression of stem cell markers *Nestin* and *KLF4* was detected by RT^2^-PCR in untreated U87-RG-GSCs, and in shU87-RG-GSCs treated with TMZ (150 µM) for 72 h. *, *p* < 0.05; ***, *p* < 0.001, compared with corresponding ev; #, *p* < 0.05; ###, *p* < 0.001, compared with evC; +, *p* < 0.05, compared with corresponding C. (**D**) Knockdown of PDCD10 enhances the viability of parental U87-RG cells and their GSC variants. U87-RG cells (left) and U87-RG-GSCs (right) received TMZ (150 M) or vehicle DMSO (0.1%) treatment for 72 h. Cell viability was detected after 72 h of treatment. *, *p* < 0.05; **, *p* < 0.01; ***, *p* < 0.001, compared with corresponding ev; #, *p* < 0.05; ##, *p* < 0.01, compared with evC; +, *p* < 0.05, ++, *p* < 0.01, compared with corresponding parental cells. (**E**) Knockdown of PDCD10 reduces the mRNA expression of MMR genes (*MSH2*, *MSH6*, and *PMS2*) in U87-RG-GSCs. U87-RG-GSCs received treatment of 150 µM TMZ or vehicle (0.1% DMSO; no treatment). Cells were harvested for PCR detection of MMR genes after 72 h of treatment. Expression of MMR genes in non-treated (**Ea**) and TMZ-treated U87-RG-GSCs (**Eb**). *, *p* < 0.05; **, *p* < 0.01; ***, *p* < 0.001, compared with ev.

GSCs, a sub-population of glioblastoma cells, have the ability to self-renew, possess tumorigenic potential, and generate differentiated tumor cells. They are responsible, at least in part, for the chemo-resistance of glioblastoma [7,9,14]. We next investigated whether shRG cells exhibit stemness traits consistent with TMZ-resistance. To determine the impact of PDCD10 knockdown on the stemness of RG cells, we carried out a neurosphere assay under serum-free conditions. Figure 8A shows the representative images of sphere formation in ev-, shU87-RG, and shU87-RG-GSCs cells. Quantitation analysis of sphere formation efficiency (SFE) demonstrated a 5.0-fold increase in SFE in shU87-RG cells, compared to in evU87-RG cells (*p* < 0.001). Moreover, compared with parental RG cells, RG-GSCs formed a higher number of spheres, and this increase was more pronounced in shRG-GSC (evRG-GSC vs. shRG-GSC = 5% vs. 24%; *p* < 0.001) (Figure 8B), suggesting that RG-GSCs possess a typical stemness feature.

We further evaluated the molecular signature of RG-GSC by detecting the expression of stem cell markers. RT^2^-PCR revealed that shRG-GSC significantly upregulated the expression of *Nestin* (3.59-fold, *p* < 0.001) and *KLF4* (2.19-fold, *p* < 0.01) (Figure 8C), compared with evRG-GSC, both under control conditions and 72 h after TMZ treatment. The results demonstrate that PDCD10 knockdown-induced stemness in U87-RG-GSC was accompanied by the increased expression of stem cell markers.

To test whether the enhanced stemness of shRG-GSC is associated with increased TMZ-resistance in our models, we compared the viability of RG cells and RG-GSC without (control, C) and with TMZ treatment. shU87-RG-GSC exhibited faster proliferation under control conditions (*p* < 0.001) and a higher viability after 72 h of TMZ treatment (*p* < 0.01) than their corresponding evU87-RG-GSC control cells. Notably, a greater TMZ-resistant effect was observed in shU87-RG-GSC, compared to their parental shU87-RG control cells (*p* < 0.01) (Figure 8D). Our data demonstrate the critical role of PDCD10 in the self-renewal of glioblastoma cells. This may, in conjunction with our data on MGMT and MMR genes, elucidate the pivotal role of PDCD10 in mediating TMZ sensitivity in glioblastoma.

Next, we investigated whether the enhanced TMZ-resistance in shRG-GSC is associated with impaired MMR facilitated by PDCD10 knockdown, as seen in parental shGBM-RG cells (Figure 6). Under untreated conditions, the basal expression levels of MMR genes *MSH2*, *MSH6*, and *PMS2* were significantly downregulated in shU87-RG-GSC, compared to in evU87-RG-GSC (Figure 8Ea). The alterations in expression levels of MMR genes were consistent in shU87-RG-GSC after TMZ treatment (Figure 8Eb).

## 4. Discussion

Tumor recurrence is an obstacle of the current GBM therapy largely attributed to acquired TMZ and radiotherapy resistance [5,6,30]. Despite efforts made over the past decades to address this issue, treatment failure manifests typically within the first 6–12 months after primary TMZ treatment [31]. This underscores the urgency to identify a key molecule that is involved in acquired TMZ-resistance.

**Figure 9:**
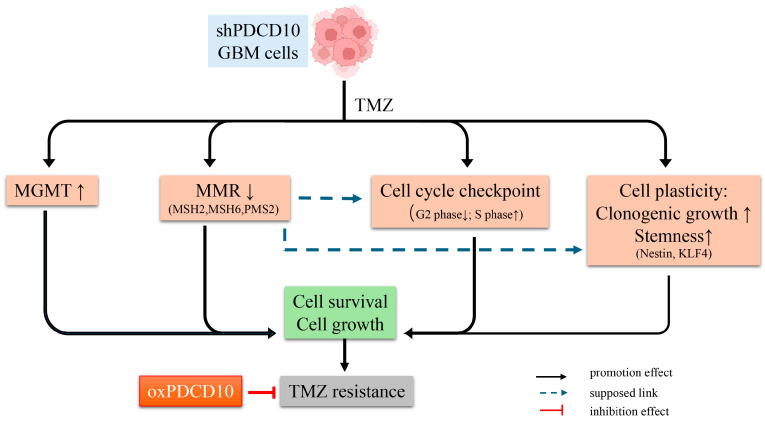
Schematic summary of the role and mechanism of PDCD10 in acquired TMZ-resistance. Knockdown of PDCD10 (shPDCD10) in GBM cells significantly increased cell survival in response to TMZ treatment, and strongly promoted tumor cell regrowth in the post-treatment phase, which collectively accounted for acquired TMZ-resistance. Mechanism studies revealed that the loss of PDCD10 modulated the expression of DNA damage response genes (i.e., upregulating MGMT and downregulating MMR genes *MSH2*, *MSH6*, and *PMS2*), and altered the cell cycle process, as evidenced by the evasion of tumor cells from arrest at the G2/M phase, and the increase in tumor cells in the proliferating S phase. In addition, shPDCD10-GBM cells exhibited higher cell plasticity, as demonstrated by an increased capacity for colony formation and transformation of shPDCD10-GBM cells into GSC-like cells that expressed higher levels of the stem cell markers Nestin and KLF4. In support of these findings, overexpression of PDCD10 (oxPDCD10) induced contrary changes in the molecular and cell behaviors observed in shPDCD10-GBM cells, increasing the sensitivity of oxPDCD10-GBM cells to TMZ treatment and suppressing tumor cell regrowth after TMZ treatment. Our results indicate that PDCD10 plays a pivotal role in acquired TMZ-resistance and thus represents a promising target for perturbing TMZ-resistance and tumor recurrence.

Here, we demonstrated that the loss of PDCD10 in GBM cells reduced sensitivity to TMZ during the treatment phase, and largely promoted the regrowth of resistant GBM cells in TMZ in the post-treatment phase. Further investigation revealed that the knockdown of PDCD10 stimulated DNA replication and proliferation by altering cell cycle checkpoints and interfering with DNA repair signaling, i.e., upregulating MGMT expression and downregulating the level of MMR genes, enabling RG cells to evade TMZ-induced cell cycle arrest. Conversely, overexpression of PDCD10 diminished all these effects in both the TMZ treatment and post-treatment phases. More interestingly, PDCD10 knockdown propagated colony formation and stemness in GBM cells in response to TMZ treatment. These GSCs derived from shPDCD10-RG cells represented stem-like cell characteristics, as evidenced by increased neurosphere formation capacity and upregulated expression of the stem cell markers Nestin and KLF4. Moreover, shPDCD10-RG-GSCs maintained an abatement of MMR expression under both TMZ no-treatment and treatment conditions, and displayed a more pronounced TMZ-resistance upon treatment, compared with the parental shPDCD10-RG cells. These data provide strong evidence for a promising role of PDCD10 in DNA repair/integrity and tumor cell plasticity, thereby contributing to TMZ-resistance (schematically summarized in Figure 9). Acquired TMZ-resistance is mostly caused by the impairment of DNA damage repair pathways, evasion from cell cycle arrest, and tumor cell stemness [5,14]. In our recently established, acute, acquired TMZ-resistance model, regrown cells (RG) that survived two cycles of TMZ treatment fully represented features of the resistant cells [13]. In the present study, we found that a loss of PDCD10 reduced the cytotoxicity of TMZ in both parental cells and in shPDCD10-RG cells in the treatment phase, but shPDCD10-RG cells exhibited a greater regrowth potency more significantly in the post-treatment phase, as demonstrated in two different regrowth models (Figure 1B and Figure 2B,C). These findings reinforce the pivotal role of PDCD10 in cell regrowth, which is highly relevant to tumor growth and eventual GBM recurrence. As supporting data, overexpression of PDCD10 increased the sensitivity of GBM cells to TMZ in the treatment phase (Figure 3B) and significantly suppressed the regrowth activity of GBM cells in the post-treatment phase (Figure 3C). These results further verify the crucial role of PDCD10 in TMZ-resistance, suggesting PDCD10 as a potential target in GBM therapy. It is worth noting that TMZ concentration used in the present study is comparable to that in other in vitro studies, however, it is not able to equal that at the tumor site in humans, which may be a limitation of laboratory research in general.

Cell cycle checkpoints act as guardians of DNA integrity and prevent the accumulation of genetic errors during cell division [32]. Bypassing cell cycle checkpoints accompanied by persistent DNA replication and uncontrolled, rapid cell proliferation significantly contribute to therapy resistance and tumor recurrence. Given the implication of PDCD10 in the proliferation and survival of GBM cells and in TMT resistance shown in our previous studies [20,26], we checked whether PDCD10 altered DNA replication and cell cycle checkpoints in RG cells in response to TMZ treatment. Cell cycle analysis showed that shPDCD10-RG cells evaded TMZ-induced cell cycle arrest in the G2/M phase but increased DNA replication in the S phase (Figure 5A,B). These cell cycle alterations directly resulted in an increased DNA replication (Figure 4A,B). Overexpression of PDCD10 did the opposite (Figure 4C,D and Figure 5C,D). Collectively, the results manifest PDCD10 as a key player in the regulation of proliferation and cell cycle checkpoints of TMZ-resistant GBM cells, and this is consistent with its previously defined role in proliferation and apoptosis in astrocytes [33], GBM cells [20,21,26], endothelial cells [34,35], and in other cancers [15,19,24,36].

MGMT is a key molecule affecting the efficacy (cytotoxicity) of TMZ by rapidly repairing O^6^-MeG lesions resulting from TMZ, thereby leading to cell survival after chemotherapy. MGMT deficiency has been associated with increased DNA double-strand breaks and greater sensitivity to TMZ treatment. CRISPR/Cas9-mediated epigenetic regulation of MGMT expression enhanced TMZ-sensitivity [37]. The present study reported that PDCD10 knockdown gradually upregulated MGMT mRNA levels 1.83-, 6.90-, and 12.33-fold in shPDCD10-RG cells in absence of TMZ (control condition), in the TMZ treatment phase, and in the post-treatment phase, respectively (Figure 6Aa), whereas overexpression of PDCD10 significantly suppressed MGMT mRNA expression (Figure 6Ab). The regulatory role of PDCD10 in MGMT expression was confirmed at the protein level (Figure 6B). To our knowledge, this is the first report on the regulatory role of PDCD10 in MGMT expression in GBM cells. It is known that promoter methylation of MGMT turns off its gene expression. Thus, MGMT methylation status in GBM tumors serves as a predictive marker for prognosis in the clinic. A recent study revealed that the methylation status of MGMT in extracellular vesicles in patient plasma is consistent with that in tumor tissue, and thus is also a usable biomarker to monitor GBM [38]. In line with these and with the inverse association of the expression of PDCD10 and MGMT defined in the present study, PDCD10 may be an alternative prognostic marker for predicting the outcome of GBM patients. To date, it is unclear how PDCD10 regulates MGMT expression in GBM cells. Of note, MGMT transcription is not only controlled by promoter methylation but also by transcription factors, such as NFκB [39]. Thus, it is highly interesting to elucidate the mechanism of PDCD10 in regulating MGMT expression, such as the axis of PDCD10/NFκB/MGMT, in the future.

MMR plays a crucial role in maintaining genome integrity by correcting mismatches during DNA replication. Impaired MMR can result in either excessively repairing or failing to repair DNA damage, thereby evading cell cycle arrest and eventually leading to uncontrolled cell growth and resistance to chemotherapy-induced cell death [5,13,40]. The present study revealed a significant downregulation of MMR genes *MSH2*, *MSH6*, and *PMS2* in shT98g-RG cells under the no treatment condition and in the TMZ treatment and post-treatment phases (Figure 6C), whereas the expression of these genes remained unchanged in PDCD10-overexpressing cells (oxT98g) under the same conditions (Figure 6D). To our knowledge, these are completely novel findings reported for the first time and are fully consistent with the effects of PDCD10 on DNA replication (Figure 4) and cell cycle checkpoints (Figure 5). So far, there is no straightforward evidence showing a direct regulatory relationship between MGMT and MMR. Thus, the PDCD10-mediated inverse correlation of MGMT and MMR gene expression is most likely achieved through distinct mechanisms. Frequent hypermutations, commonly associated with mutations in MMR genes, have been found in recurrent tumors [41]. How PDCD10 regulates these DDR genes in GBM cells is a topic of great interest that requires further study in the future.

GSCs play a critical role in clonal expansion and the development of therapy-resistant GBM subclones. Tumor cells may show some plasticity, undergoing dedifferentiation into more pluripotent progenitor cells under certain conditions [42,43]. TMZ stimulates the transformation of differentiated tumor cells into GSCs that express pluripotency markers and exhibit increased tumor-initiating capabilities. Thus, genomic instability may enhance cellular plasticity and tumor heterogeneity, providing a pool of diverse cells including GSCs [9,44]. Based on the findings regarding the role of PDCD10 in genotypic alterations of DNA damage-repair genes and subsequent changes in cell behavior leading to TMZ-resistance, we questioned whether shPDCD10 induced the transdifferentiation of GBM cells into GSC-like cells. In this context, we found that shPDCD10-RG cells exhibited a remarkable increased clonogenic growth capacity (Figure 7) and formed more spheres under serum-free conditions (Figure 8). shPDCD10-RG-derived GSCs showed greater resistance to TMZ, comparable with parental shPDCD10-RG cells, accompanied by high expression of Nestin and KLF4 (Figure 8), which are markers of pro-neural GSC [44,45,46].

It is worth mentioning that both Nestin and KLF4 not only serve as GSC markers but also have their own distinct functions. Nestin is regulated by Notch signaling that is activated in GBM [46,47], and implicated in GBM progression and angiogenesis in association with several downstream pathways, including PI3K/Akt and Erk [21,47]. KLF4, a zinc finger transcription factor, plays a crucial role in the pathogenesis of inflammation and tumorigenesis [48]. In GBM, KLF4 has been shown to activate Notch and its downstream target Nestin, thereby establishing proneural GSC identity and contributing to the survival of GSCs and tumor growth [49,50]. High levels of KLF4 expression have been associated with poor outcomes in GBM [49]. Interestingly, it has been shown that PDCD10-depleted endothelial cells (*Ccm3*-null endothelial cells) express mesenchymal/stem-cell markers, including KLF4, and induce the transdiffrentiation to mesenchymal-like cells. Thus, these PDCD10-depleted cells are reminiscent of the tumor-initiating cells that are responsible for tumor growth [51]. Our data, together with this report, indicate that KLF4 is upregulated in PDCD10-deficient cells and is a key molecule involved in the regulation of cell plasticity. Taken together, compelling findings from our study suggest that the loss of PDCD10 facilitates the dedifferentiation of TMZ-resistant cells (RG cells) into stem-like cells that could serve as cells of origin in recurrent tumors.

## 5. Conclusions

Using transduced PDCD10-knockdown and -overexpressing GBM cells, the present study provided strong evidence for a promising role of PDCD10 in governing the sensitivity of GBM cells during TMZ treatment and in promoting cell regrowth post-TMZ treatment, which are representative of chemo-resistance and tumor relapse in clinical settings, respectively. Furthermore, we reported for the first time that PDCD10 regulates the expression of DDR genes (MGMT and MMR genes), and modulates the cell cycle checkpoint, which crucially contributes to TMZ-resistance and cell regrowth. Notably, knockdown of PDCD10 induced cell plasticity, transforming PDCD10 knockdown cells into GSCs that exhibited increased resistance to TMZ. The PDCD10 knockdown cell-derived GSCs may, thus, serve as the origin of TMZ-resistant and rapidly regrowing cells, accounting for tumor recurrence. Overall, the present study underscores the significant impact of PDCD10 on TMZ-resistance and GBM recurrence, suggesting PDCD10 as a novel target for GBM prognosis and therapy.

## Figures and Tables

**Table 1:** List of primers and corresponding annealing temperatures for RT^2^-PCR.

Primer Name	Sequence	Annealing Temperature (°C)
*PDCD10*		60
for	TGGCAGCTGATGATGTAGAAG	
rev	TCGTGCCTTTTCGTTTAGGT	
*MGMT*		60
for	ACCGTTTGCGACTTGGTACTT	
rev	GGAGCTTTATTTCGTGCAGACC	
*MSH2*		60
for	TTTACCCGGAGGAGAGACTGC	
rev	TGCTCTCCCTTTTTGCCTTTC	
*MSH6*		60
for	AGAGCAATGCAACGTGCAGA	
rev	TTTGGCGGCTACTTCGCCTA	
*PMS2*		60
for	ATCGGCGAAGGTTGGAACTC	
rev	CGGATGCCTGCTGAAATGAT	
*Nestin*		60
for	CTCCAAGAATGGAGGCTGTAGGAA	
rev	CCTATGAGATGGAGCAGGCAAGA	
*KLF4*		60
for	GGCTGCGGCAAAACCTACAC	
rev	CGGGCGAATTTCCATCCAC	
*GAPDH*		60
for	TCACCACCATGGAGAAGGC	
rev	GCTAAGCAGTTGGTGGTGCA	
*RPS13*		60
for	CGAAAGCATCTTGAGAGGAACA	
rev	TCGAGCCAAACGGTGAATC	

for: forward; rev: reverse.

## Data Availability

The original contributions presented in the study are included in the article/Supplementary Material, further inquiries can be directed to the corresponding author.

## Supplementary Materials

The following supporting information can be downloaded at: https://www.mdpi.com/article/10.3390/cells13171442/s1, Figure S1. Knockdown of PDCD10 enables U87-RG cells to sustain DNA replication and attenuate a cell cycle arrest in response to TMZ treatment; Figure S2. EdU incorporation assay of ev/shT98g-RG and ev/oxT98g cells treated with DMSO; Figure S3. Cell cycle assay of ev/shT98g-RG and ev/oxT98g cells treated with DMSO; Figure S4. Sub-G1 analysis of ev/shT98g-RG and ev/oxT98g cells 72 h after TMZ treatment and 3 d of regrowth; Figure S5. Original immunoblots for Figure 1Ab; Figure S6. Original immunoblots for Figure 3Ab; Figure S7. Original immunoblots for Figure 6Ba.
