# Supplementary material for: PDCD10 Is a Key Player in TMZ-Resistance and Tumor Cell Regrowth: Insights into Its Underlying Mechanism in Glioblastoma Cells
Source: Cells. 2024 Aug 28;13(17):1442. doi: 10.3390/cells13171442 (PMC11394141; doi:10.3390/cells13171442)
Supplement: Supplementary file 1 [file cells-13-01442-s001.zip › cells-3164773-supplementary.pptx]

## Slide 1
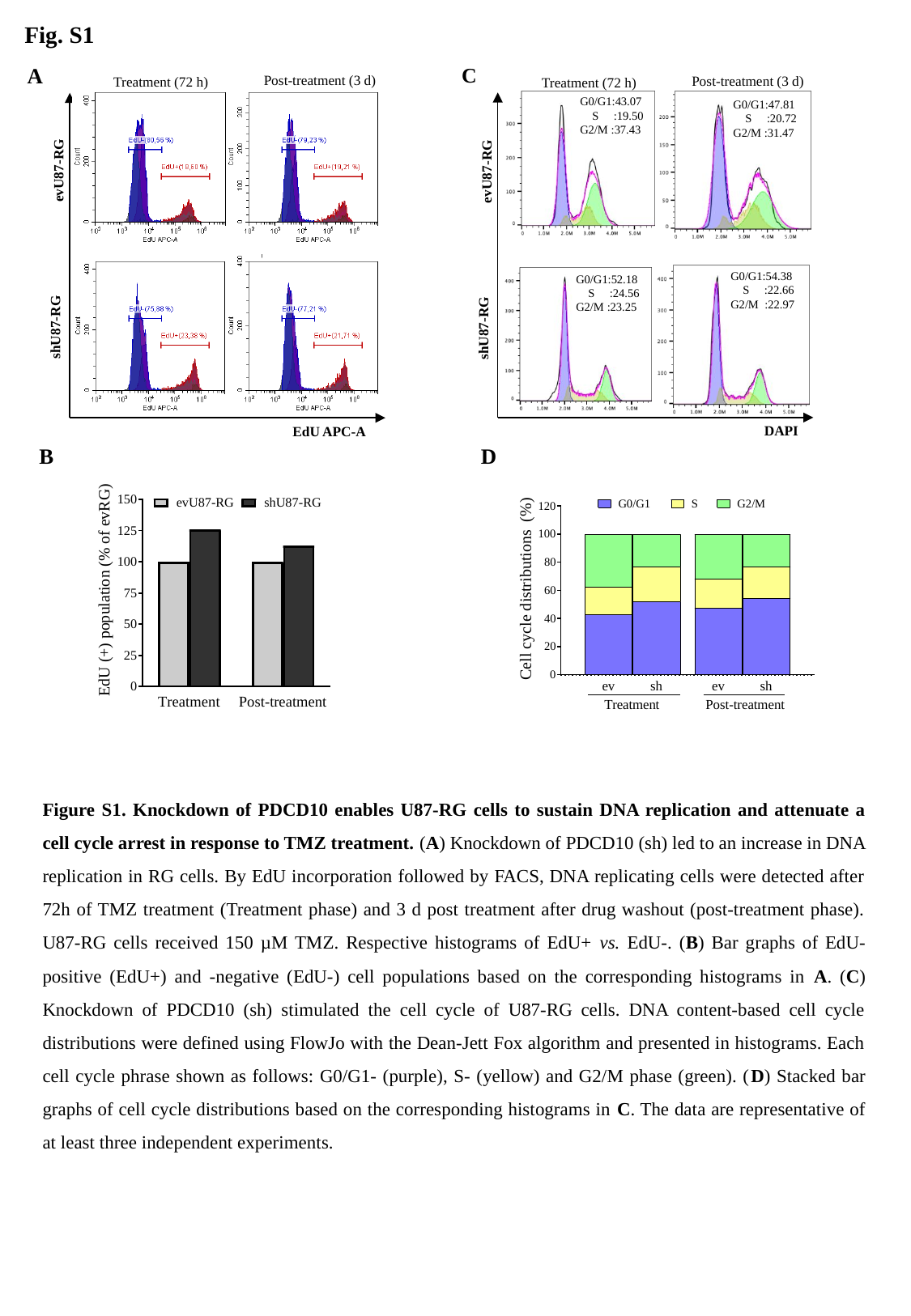

Fig. S1
A
C
Post-treatment (3 d)
Post-treatment (3 d)
Treatment (72 h)
Treatment (72 h)
G0/G1:47.81
 S :20.72
G2/M :31.47
G0/G1:43.07
 S :19.50
G2/M :37.43
G0/G1:54.38
 S :22.66
G2/M :22.97
G0/G1:52.18
 S :24.56
G2/M :23.25
 evU87-RG
 evU87-RG
 shU87-RG
 shU87-RG
DAPI
EdU APC-A
D
B
Figure S1. Knockdown of PDCD10 enables U87-RG cells to sustain DNA replication and attenuate a cell cycle arrest in response to TMZ treatment. (A) Knockdown of PDCD10 (sh) led to an increase in DNA replication in RG cells. By EdU incorporation followed by FACS, DNA replicating cells were detected after 72h of TMZ treatment (Treatment phase) and 3 d post treatment after drug washout (post-treatment phase). U87-RG cells received 150 µM TMZ. Respective histograms of EdU+ vs. EdU-. (B) Bar graphs of EdU-positive (EdU+) and -negative (EdU-) cell populations based on the corresponding histograms in A. (C) Knockdown of PDCD10 (sh) stimulated the cell cycle of U87-RG cells. DNA content-based cell cycle distributions were defined using FlowJo with the Dean-Jett Fox algorithm and presented in histograms. Each cell cycle phrase shown as follows: G0/G1- (purple), S- (yellow) and G2/M phase (green). (D) Stacked bar graphs of cell cycle distributions based on the corresponding histograms in C. The data are representative of at least three independent experiments.

## Slide 2
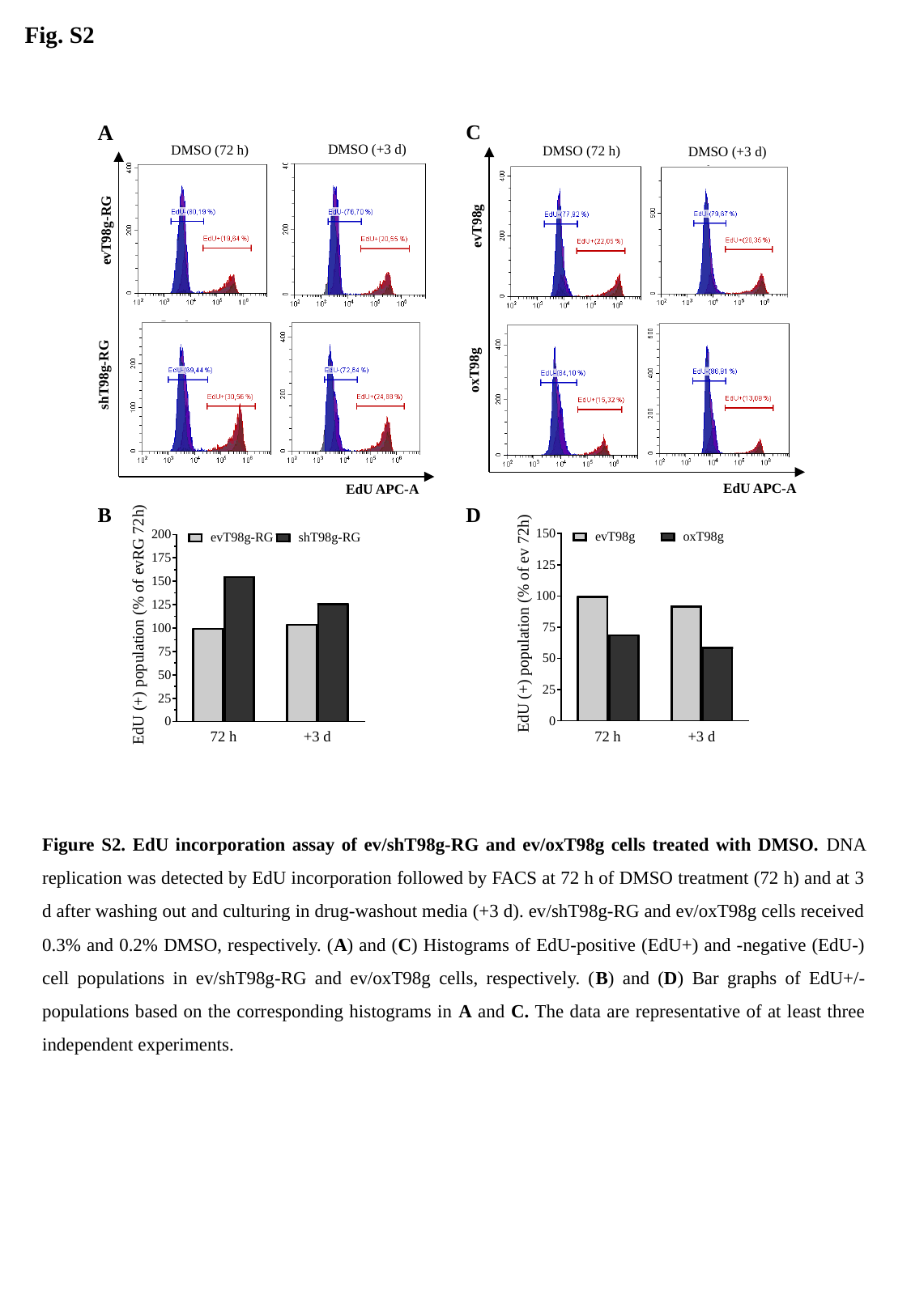

Fig. S2
A
C
DMSO (+3 d)
DMSO (72 h)
DMSO (72 h)
DMSO (+3 d)
 evT98g
 evT98g-RG
 oxT98g
 shT98g-RG
EdU APC-A
EdU APC-A
B
D
Figure S2. EdU incorporation assay of ev/shT98g-RG and ev/oxT98g cells treated with DMSO. DNA replication was detected by EdU incorporation followed by FACS at 72 h of DMSO treatment (72 h) and at 3 d after washing out and culturing in drug-washout media (+3 d). ev/shT98g-RG and ev/oxT98g cells received 0.3% and 0.2% DMSO, respectively. (A) and (C) Histograms of EdU-positive (EdU+) and -negative (EdU-) cell populations in ev/shT98g-RG and ev/oxT98g cells, respectively. (B) and (D) Bar graphs of EdU+/- populations based on the corresponding histograms in A and C. The data are representative of at least three independent experiments.

## Slide 3
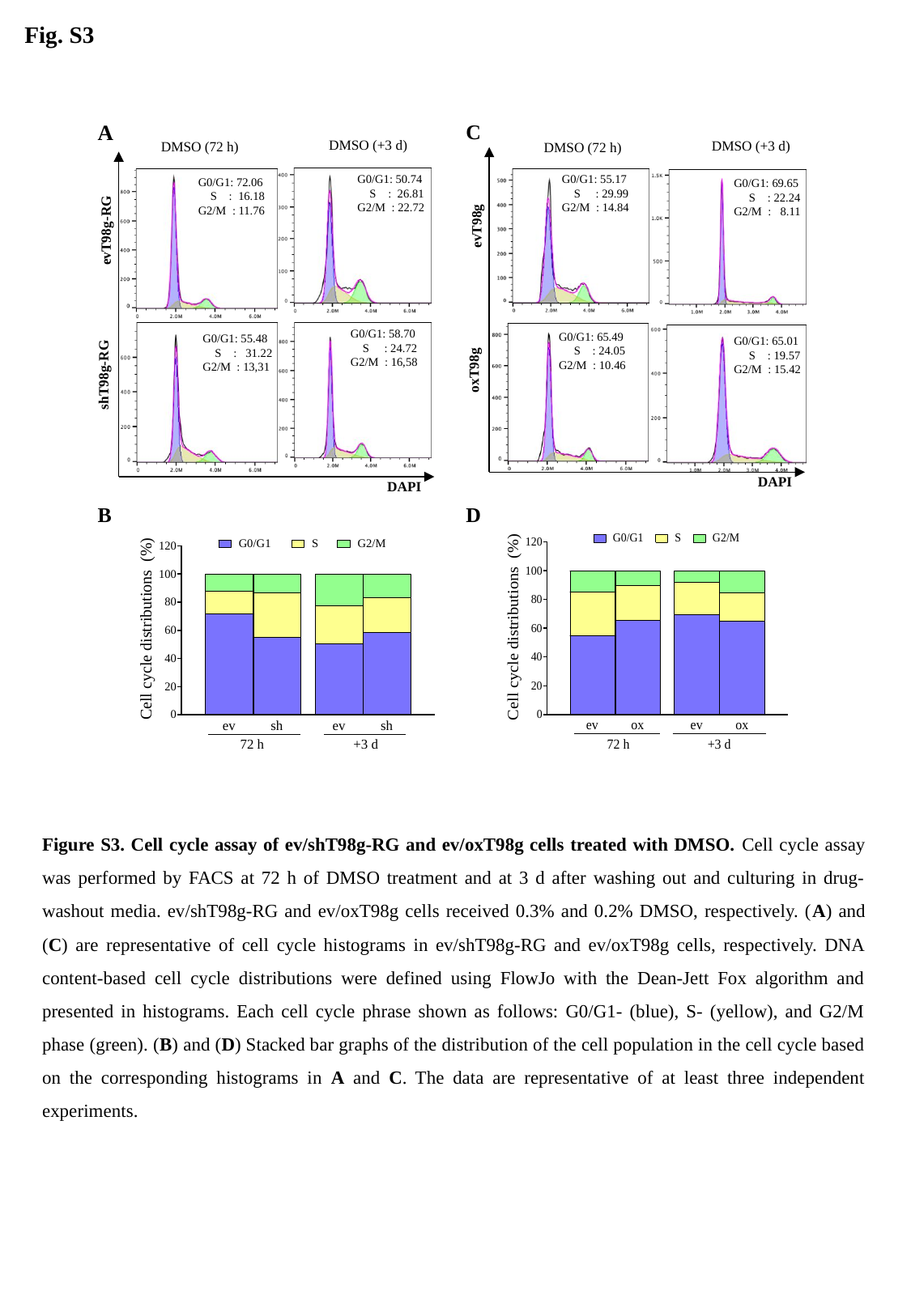

Fig. S3
A
C
DMSO (+3 d)
DMSO (+3 d)
DMSO (72 h)
DMSO (72 h)
G0/G1: 50.74
 S : 26.81
G2/M : 22.72
G0/G1: 55.17
 S : 29.99
G2/M : 14.84
G0/G1: 72.06
 S : 16.18
G2/M : 11.76
G0/G1: 69.65
 S : 22.24
G2/M : 8.11
 evT98g
 evT98g-RG
G0/G1: 58.70
 S : 24.72
G2/M : 16,58
G0/G1: 55.48
 S : 31.22
G2/M : 13,31
G0/G1: 65.49
 S : 24.05
G2/M : 10.46
G0/G1: 65.01
 S : 19.57
G2/M : 15.42
 oxT98g
 shT98g-RG
DAPI
DAPI
B
D
Figure S3. Cell cycle assay of ev/shT98g-RG and ev/oxT98g cells treated with DMSO. Cell cycle assay was performed by FACS at 72 h of DMSO treatment and at 3 d after washing out and culturing in drug-washout media. ev/shT98g-RG and ev/oxT98g cells received 0.3% and 0.2% DMSO, respectively. (A) and (C) are representative of cell cycle histograms in ev/shT98g-RG and ev/oxT98g cells, respectively. DNA content-based cell cycle distributions were defined using FlowJo with the Dean-Jett Fox algorithm and presented in histograms. Each cell cycle phrase shown as follows: G0/G1- (blue), S- (yellow), and G2/M phase (green). (B) and (D) Stacked bar graphs of the distribution of the cell population in the cell cycle based on the corresponding histograms in A and C. The data are representative of at least three independent experiments.

## Slide 4
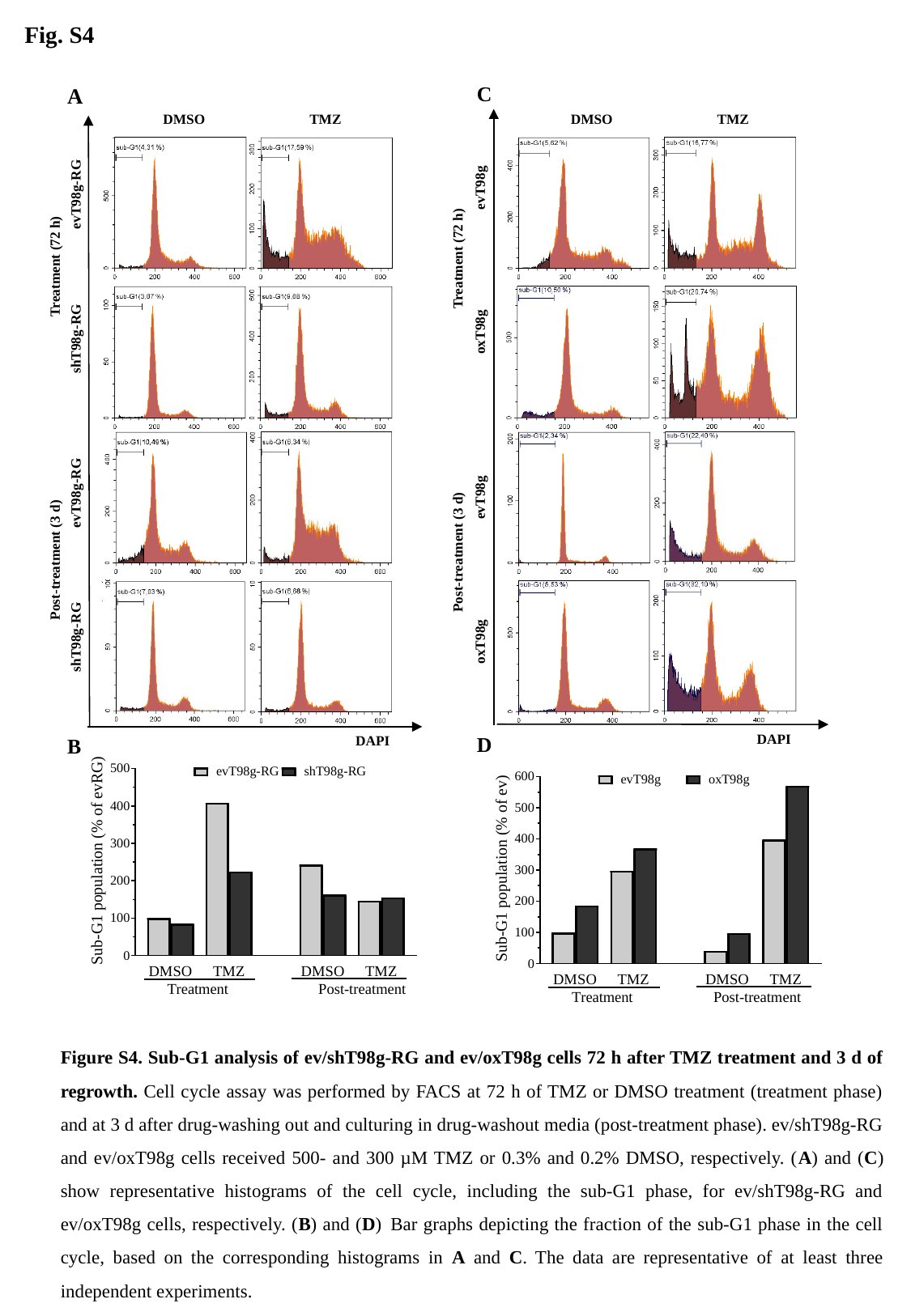

Fig. S4
C
A
DMSO
TMZ
DMSO
TMZ
 evT98g
 evT98g-RG
Treatment (72 h)
Treatment (72 h)
 oxT98g
 shT98g-RG
 evT98g-RG
 evT98g
Post-treatment (3 d)
Post-treatment (3 d)
 shT98g-RG
 oxT98g
DAPI
D
DAPI
B
Figure S4. Sub-G1 analysis of ev/shT98g-RG and ev/oxT98g cells 72 h after TMZ treatment and 3 d of regrowth. Cell cycle assay was performed by FACS at 72 h of TMZ or DMSO treatment (treatment phase) and at 3 d after drug-washing out and culturing in drug-washout media (post-treatment phase). ev/shT98g-RG and ev/oxT98g cells received 500- and 300 µM TMZ or 0.3% and 0.2% DMSO, respectively. (A) and (C) show representative histograms of the cell cycle, including the sub-G1 phase, for ev/shT98g-RG and ev/oxT98g cells, respectively. (B) and (D)  Bar graphs depicting the fraction of the sub-G1 phase in the cell cycle, based on the corresponding histograms in A and C. The data are representative of at least three independent experiments.

## Slide 5
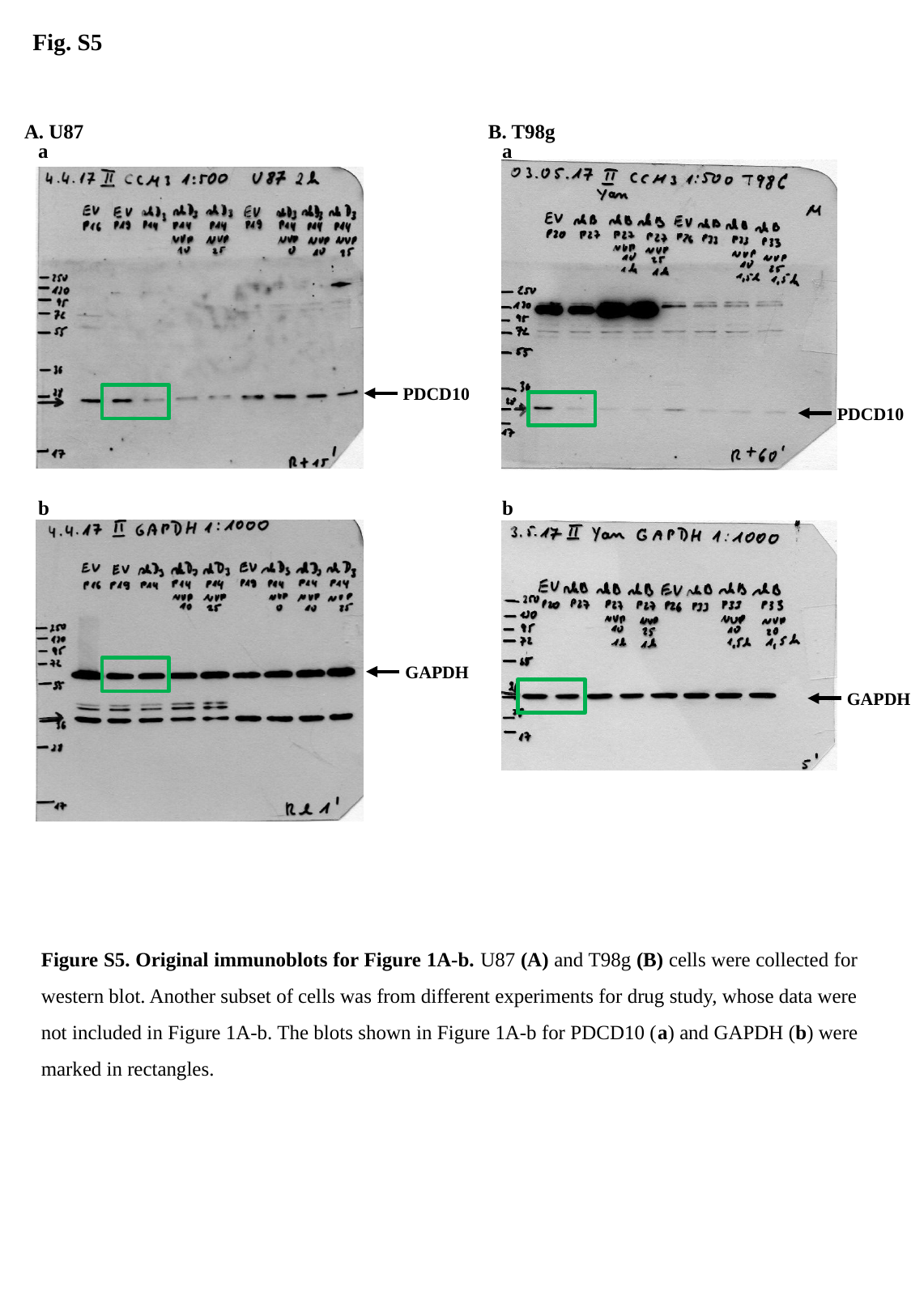

Fig. S5
A. U87
B. T98g
a
a
PDCD10
PDCD10
b
b
GAPDH
GAPDH
Figure S5. Original immunoblots for Figure 1A-b. U87 (A) and T98g (B) cells were collected for western blot. Another subset of cells was from different experiments for drug study, whose data were not included in Figure 1A-b. The blots shown in Figure 1A-b for PDCD10 (a) and GAPDH (b) were marked in rectangles.

## Slide 6
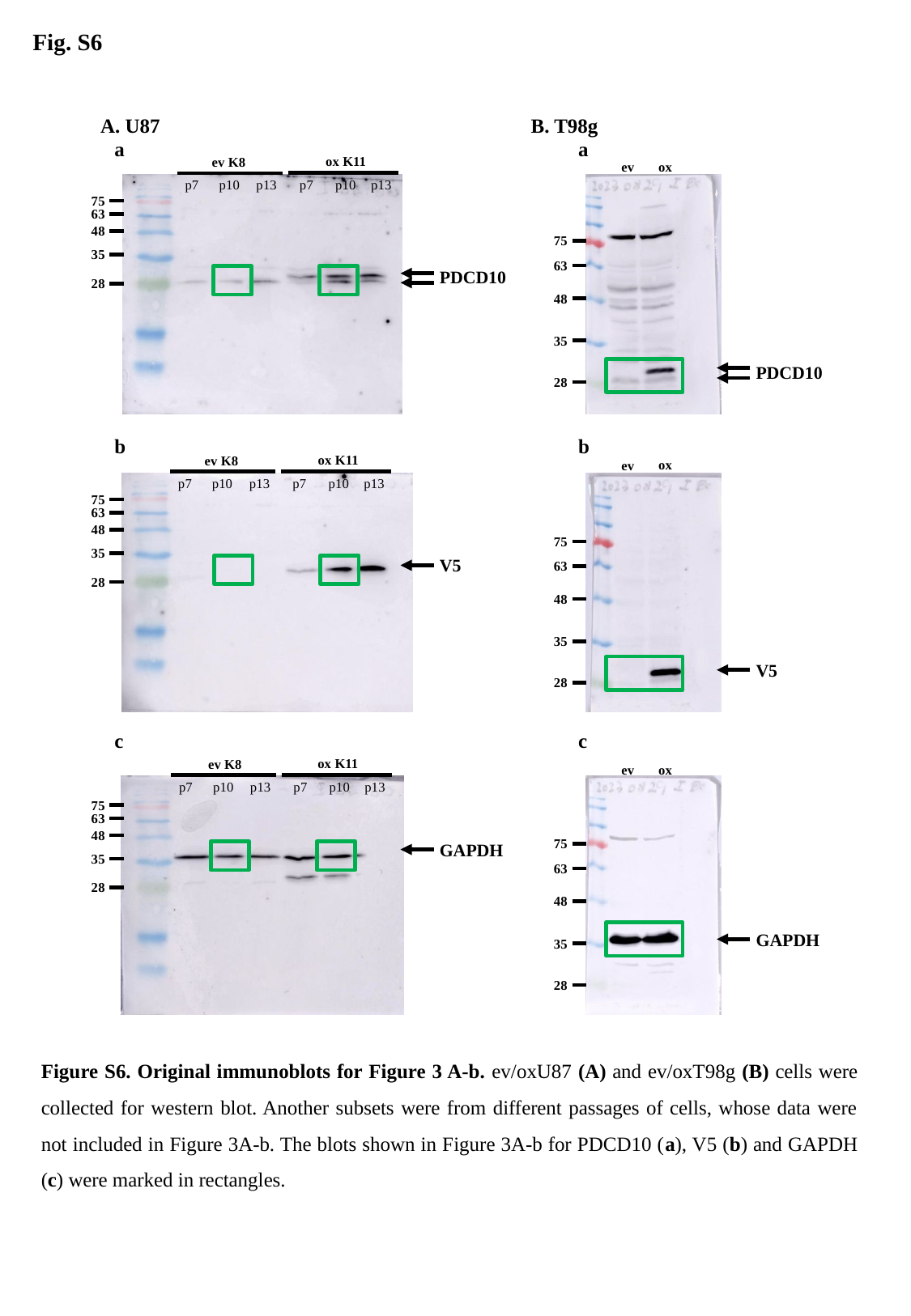

Fig. S6
A. U87
B. T98g
a
a
ox K11
ev K8
p7
p10
p13
p7
p10
p13
ox
ev
75
63
48
35
28
75
63
48
35
28
PDCD10
PDCD10
b
b
ox K11
ev K8
p7
p10
p13
p7
p10
p13
ox
ev
75
63
48
35
28
75
63
48
35
28
V5
V5
c
c
ox K11
ev K8
p7
p10
p13
p7
p10
p13
ox
ev
75
63
48
35
28
75
63
48
35
28
GAPDH
GAPDH
Figure S6. Original immunoblots for Figure 3 A-b. ev/oxU87 (A) and ev/oxT98g (B) cells were collected for western blot. Another subsets were from different passages of cells, whose data were not included in Figure 3A-b. The blots shown in Figure 3A-b for PDCD10 (a), V5 (b) and GAPDH (c) were marked in rectangles.

## Slide 7
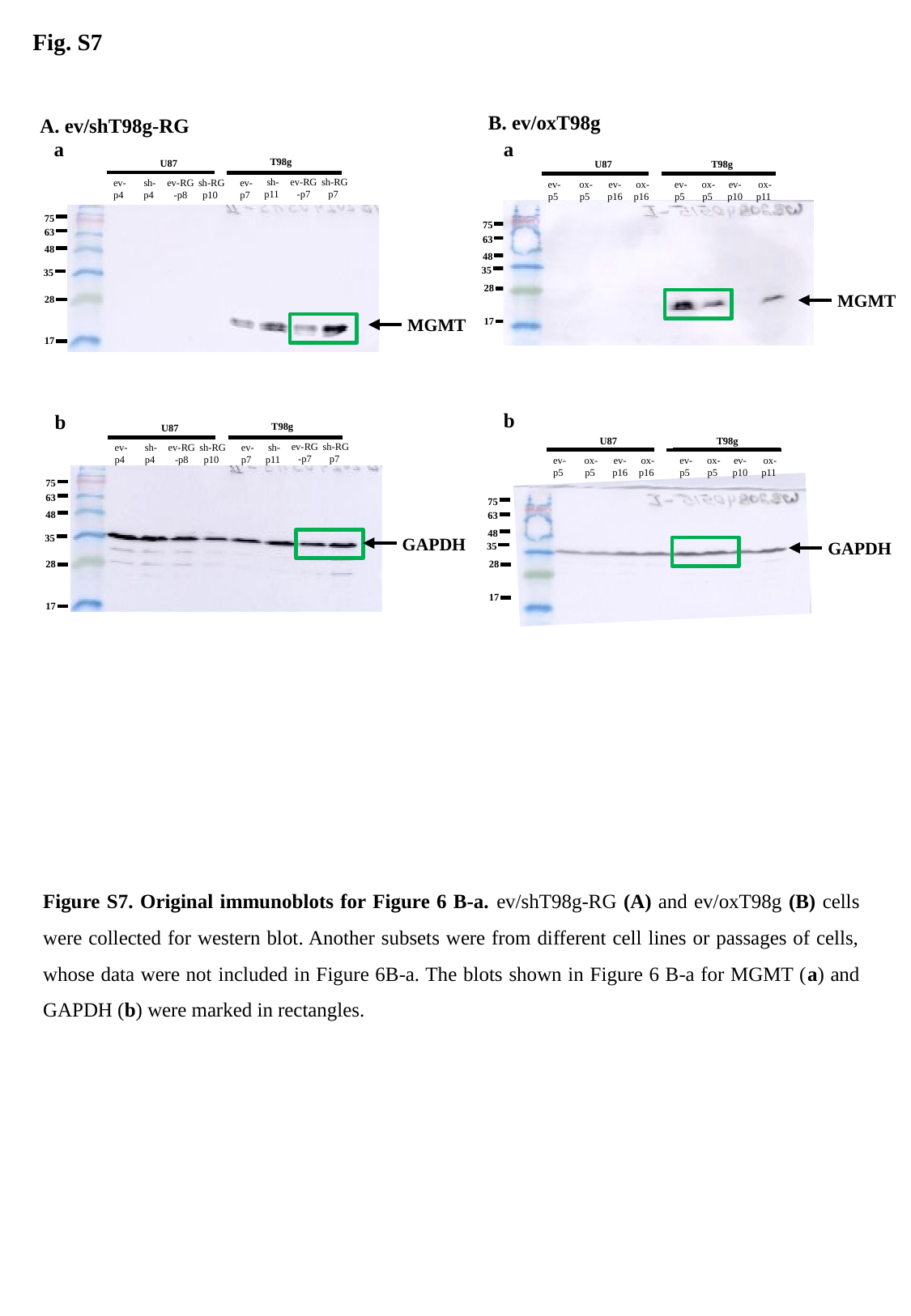

Fig. S7
B. ev/oxT98g
A. ev/shT98g-RG
a
a
T98g
U87
 sh-RG
p7
ev-RG
-p7
 sh-p11
 sh-RG
p10
 ev-
p4
ev-RG
-p8
 ev-
p7
 sh-p4
75
63
48
35
28
17
U87
T98g
 ev-
p5
 ox-p5
ev-
p16
 ox-
p16
 ev-
p5
 ox-p5
ev-
p10
 ox-
p11
75
63
48
35
28
17
MGMT
MGMT
b
b
T98g
U87
 sh-RG
p7
ev-RG
-p7
 sh-p11
 sh-RG
p10
 ev-
p4
ev-RG
-p8
 ev-
p7
 sh-p4
75
63
48
35
28
17
U87
T98g
 ev-
p5
 ox-p5
ev-
p16
 ox-
p16
 ev-
p5
 ox-p5
ev-
p10
 ox-
p11
75
63
48
35
28
17
GAPDH
GAPDH
Figure S7. Original immunoblots for Figure 6 B-a. ev/shT98g-RG (A) and ev/oxT98g (B) cells were collected for western blot. Another subsets were from different cell lines or passages of cells, whose data were not included in Figure 6B-a. The blots shown in Figure 6 B-a for MGMT (a) and GAPDH (b) were marked in rectangles.
